# Inhibition of DAMP actions in the tumoral microenvironment using lactoferrin-glycyrrhizin conjugate for glioblastoma therapy

**DOI:** 10.1186/s40824-023-00391-w

**Published:** 2023-05-20

**Authors:** Hyung Shik Kim, Seok Chan Park, Hae Jin Kim, Dong Yun Lee

**Affiliations:** 1 Department of Bioengineering, College of Engineering, and BK FOUR Biopharmaceutical Innovation Leader for Education and Research Group, Institute of Nano Science and Technology (INST), Hanyang University, and Elixir Pharmatech Inc, 222 Wangsimni-Ro, Seongdong-Gu, Seoul, 04763 Republic of Korea; 2 Institute of Nano Science and Technology (INST) & Institute For Bioengineering and Biopharmaceutical Research (IBBR), Hanyang University, Seoul, 04763 Republic of Korea; 3 Elixir Pharmatech Inc., Seoul, 07463 Republic of Korea

**Keywords:** Damaged-associated molecular patterns, Glioblastoma multiforme, Tumor-microenvironment, Glycyrrhizin, Lactoferrin

## Abstract

**Background:**

High-mobility group box-1 (HMGB1) released from the tumor microenvironment plays a pivotal role in the tumor progression. HMGB1 serves as a damaged-associated molecular pattern (DAMP) that induces tumor angiogenesis and its development. Glycyrrhizin (GL) is an effective intracellular antagonist of tumor released HMGB1, but its pharmacokinetics (PK) and delivery to tumor site is deficient. To address this shortcoming, we developed lactoferrin-glycyrrhizin (Lf-GL) conjugate.

**Methods:**

Biomolecular interaction between Lf-GL and HMGB1 was evaluated by surface plasmon resonance (SPR) binding affinity assay. Inhibition of tumor angiogenesis and development by Lf-GL attenuating HMGB1 action in the tumor microenvironment was comprehensively evaluated through in vitro, ex vivo, and in vivo. Pharmacokinetic study and anti-tumor effects of Lf-GL were investigated in orthotopic glioblastoma mice model.

**Results:**

Lf-GL interacts with lactoferrin receptor (LfR) expressed on BBB and GBM, therefore, efficiently inhibits HMGB1 in both the cytoplasmic and extracellular regions of tumors. Regarding the tumor microenvironment, Lf-GL inhibits angiogenesis and tumor growth by blocking HMGB1 released from necrotic tumors and preventing recruitment of vascular endothelial cells. In addition, Lf-GL improved the PK properties of GL approximately tenfold in the GBM mouse model and reduced tumor growth by 32%. Concurrently, various biomarkers for tumor were radically diminished.

**Conclusion:**

Collectively, our study demonstrates a close association between HMGB1 and tumor progression, suggesting Lf-GL as a potential strategy for coping with DAMP-related tumor microenvironment.

**Graphical Abstract:**

HMGB1 is a tumor-promoting DAMP in the tumor microenvironment. The high binding capability of Lf-GL to HMGB1 inhibits tumor progression cascade such as tumor angiogenesis, development, and metastasis. Lf-GL targets GBM through interaction with LfR and allows to arrest HMGB1 released from the tumor microenvironment. Therefore, Lf-GL can be a GBM treatment by modulating HMGB1 activity.

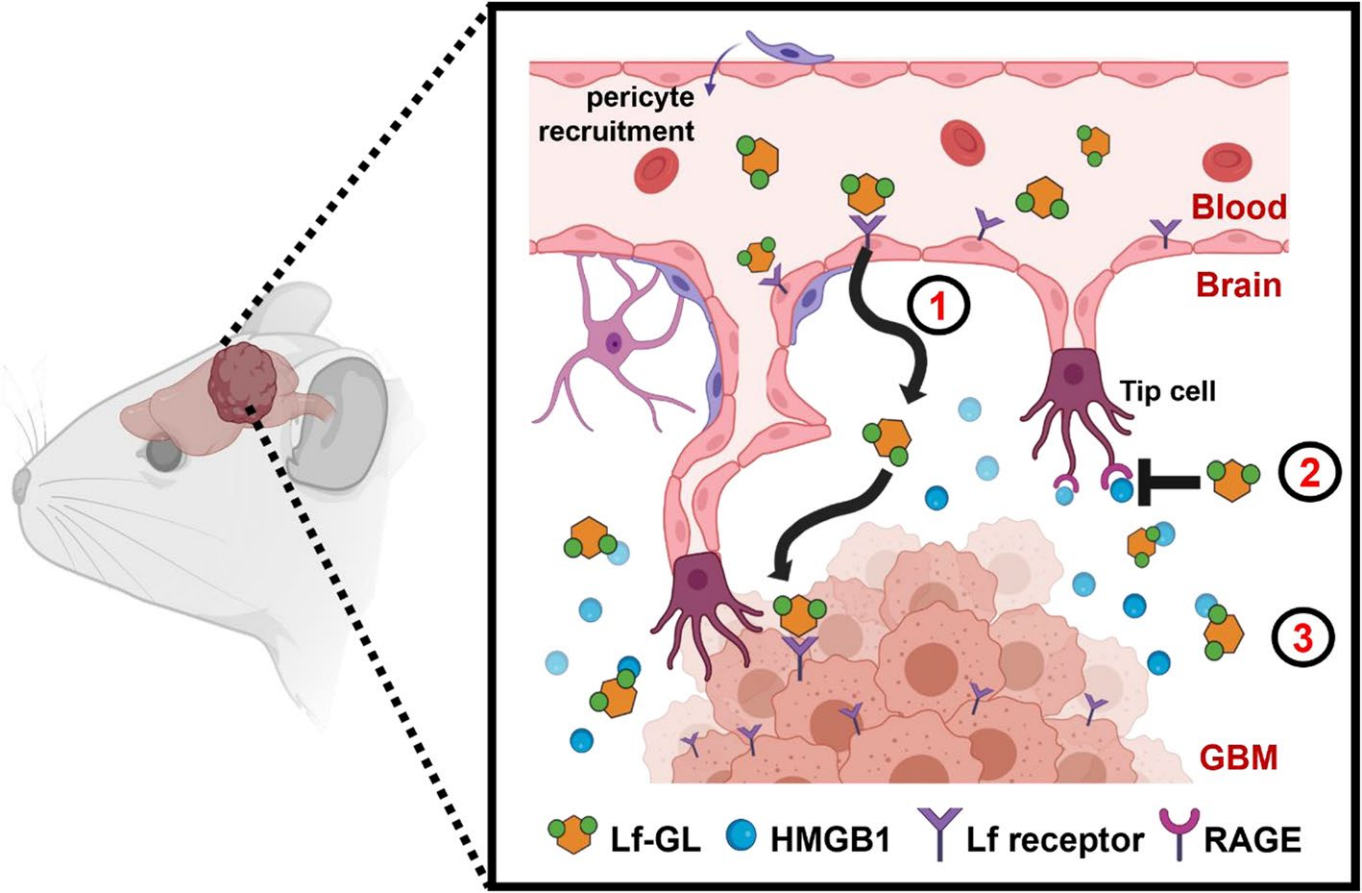

**Supplementary Information:**

The online version contains supplementary material available at 10.1186/s40824-023-00391-w.

## Background

Glioblastoma multiforme (GBM) is the most common primary malignant brain tumor, and the median survival is less than a year after diagnosis [1]. Current standards of care include surgical resection followed by radiation therapy and chemotherapy, but only 3 to 5% of GBM patient survive beyond 5 years [2]. In general, GBM tissues are characterized by high expression levels of microvascular proliferation due to upregulation of pro-angiogenesis cytokines [3]. Therefore, anti-angiogenesis has been a priority strategy of drug development for GBM over the last decade. Angiogenesis is intricately linked with tumor progression due to a lack of nutrients and oxygen. In the tumor microenvironment, abnormal and functionally immature blood vessels are generated due to dysregulated factors such as angiogenesis inhibitors, angiogenic growth factors, and other molecules that may act as angiogenesis mediators [4]. Vascular endothelial growth factor (VEGF) is the most representative and well-known angiogenesis regulator in tumors that induces angiogenic intracellular signaling through the mediation of receptor kinases that stimulate pro-angiogenic activity and endothelial cell migration [5]. Bevacizumab, a recombinant monoclonal antibody targeting VEGF ligand A, had received accelerated approval for recurrent GBM in United States, but has not shown any survival benefit in newly diagnosed GBM patients in multiple large-scale phase 3 clinical trials [6]. Therefore, the search for novel angiogenesis modulators and the pursuit of alternative GBM anti-angiogenic therapies have been demanded to date.

High-mobility group box-1 (HMGB1) is a ubiquitous non-histone nuclear protein that acts as an architectural chromatin binding factor [7]. However, once transported into cytoplasm or extracellular space, it participates as a damage-associated molecular patterns (DAMP) that binds to the receptor for advanced glycation end products (RAGE) [8]. Subsequently, it activates AKT and ERK signaling pathways and promotes tumor cell invasion [9, 10]. Recent clinical studies have shown that overexpressed HMGB1 is involved in GBM development, and its expression levels are closely related to the advanced stage of GBM and poor prognosis [11]. HMGB1 released from necrotic tumor cells, a natural phenomenon in tumor development, is expected to be highly involved in angiogenesis and tumor cell progression. Consistent with that expectation, either actively secreted or passively released HMGB1 has been reported to regulate angiogenesis, including promoting endothelial progenitor cells, homing them to tumor tissues, and inducing endothelial cell sprouting and migration [12]. Given the tumor microenvironment, blockade therapy of HMGB1 binding to RAGE is considered a potent strategy to concurrently inhibit angiogenesis and tumor progression. Therefore, delivery of HMGB1-specific antagonists to the tumor microenvironment can efficiently inhibit tumorigenic, angiogenesis and GBM development.

Glycyrrhizin (GL), the active compound of licorice, is a direct antagonist of HMGB1. The inhibitory effect of GL on HMGB1 is manifested in two aspects [13]. GL blocks the release of HMGB1 into the extracellular space by interacting with the two shallow concave surfaces formed by the two arms of the HMG boxes. Also, it inhibits HMGB1 phosphorylation inside the cell, thereby reducing the secretion of HMGB1 and decreasing its overall expression. In addition, GL is found to suppress angiogenic activities of endothelial cells by downregulating ERK angiogenic signaling pathway [14], and also elicits anti-tumor effects by downregulating the NF-κB signaling [15]. Therefore, considering the tumor microenvironment in which tumors deteriorate through HMGB1, GL, which has both antitumor and HMGB1 inhibitory effects, could play a pivotal role in GBM treatment. However, GL has poor bioavailability due to short biological half-life and lacks the GBM targeting ability, which hinders its clinical application. Therefore, the drug delivery system is required for its therapeutic application to GBM.

**Fig. 1:**
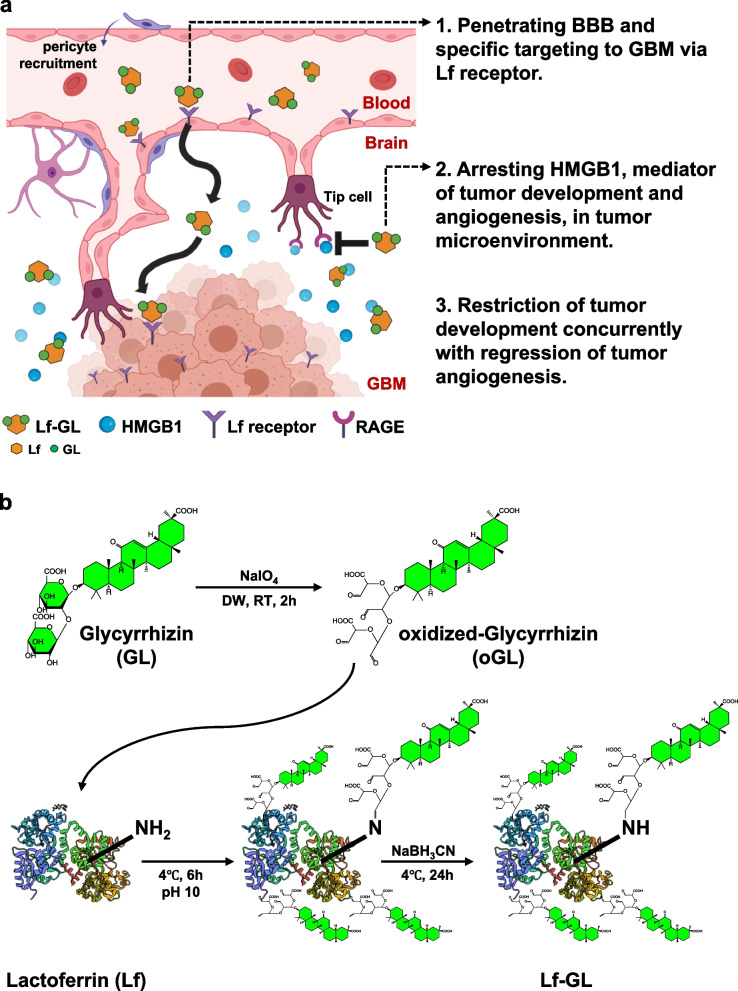
Schematic illustration and synthetic procedure for Lf-GL. **a** In the tumor microenvironment, HMGB1 secreted from GBM cells binds to RAGE and is involved in cancer development and angiogenesis. Lf-GL delivered to the tumor microenvironment through interactions with LfR overexpressed in BBB and GBM cells inhibits its activity with high binding affinity to HMGB1. Thereby, Lf-GL can inhibit GBM-mediated angiogenesis activated by HMGB1 while limiting tumor development. **b** Synthetic procedure of Lf-GL conjugation. NaIO4 refers to sodium periodate; NaBH_3_CN refers to cyanoborohydride; text under arrow refers to synthesis conditions. The illustration was created with BioRender.com

Our previous studies demonstrated that the modification of drugs via lactoferrin (Lf) can target GBM by penetrating the blood–brain barrier (BBB) and increasing their stability and half-life of blood circulation [16, 17]. This was also possible because the lactoferrin receptor (LfR) is highly expressed in the blood–brain barrier (BBB) and GBM cells and on brain endothelial cells [18–21]. Moreover, Lf, a product of exocrine glands that found in many parts of the human body, prevents the partial first-pass effects of hepatic metabolism and allow it to persist longer [22]. Therefore, to overcome the limitations of GL for GBM targeting, here we introduced lactoferrin (Lf) as a targeting ligand for GL delivery. Collectively, Lf-GL conjugate was delivered to GBM, and it effectively regulated the tumorigenic activity of HMGB1 in the tumor microenvironment (Fig. 1a).

## Materials and methods

### Materials

Glycyrrhizin (ammonium salt, purity 95%), sodium periodate (NaIO_4_), lactoferrin human (Lf), sodium carbonate (Na_2_CO_3_), sodium bicarbonate (NaHCO_3_), sodium cyanoborohydride (NaBH_3_CN), fluorescein isothiocyanate isomer 1 (FITC), 2-mercaptoethanol and tetramethylethylenediamine (TEMED), and water (HPLC grade) wer.e purchased from Sigma-Aldrich (St. Louis, MO, USA). 4′,6-diamidino-2-phenylindole (DAPI) was purchased from Vector Laboratories (Burlingame, CA, USA). NP40, cell extraction buffer, and Lysotracker® were purchased from Invitrogen (Waltham, MA, USA). Phenylmethylsulfonyl fluoride (PMSF), acetonitrile, and methanol (HPLC grade) were purchased from Thermo Fisher Scientific (Waltham, MA, USA). Paraformaldehyde (4%) was purchased by Wako (Kanto, Saitama, Japan). Protease inhibitor and Calcein AM (Cat #: BMD00064) were purchased from Abbkine (Wuhan, China). Sodium dodecyl sulfate was purchased from Affymetrix (Waltham, MA, USA). Acrylamide solution (30%) and ammonium persulfate (APS) were purchased by BIO-RAD (Hercules, CA, USA). Coomassie blue solution was purchased from Abcam (Cambridge, UK).

### Experimental cell lines and animals

In vitro experiments were performed using the U87MG human glioma cell line (Korean Cell Line Bank, Seoul, Korea), human umbilical vein endothelial cell line (HUVEC; LONZA, NJ, USA), and the human epithelial colorectal cell line (Caco-2; Korean Cell Line Bank, Seoul, Korea). U87MG (passage numbers 2 to 5) and Caco-2 cells were cultured using Dulbecco’s modified Eagle’s medium (DMEM; GenDEPOT, TX, USA) containing 10% fetal bovine serum (FBS; GenDEPOT), 1% penicillin–streptomycin (Thermo Fisher Scientific) in standard culture conditions at 37 °C and 5% CO_2_. HUVECs (passage numbers 2 to 4) were cultured using endothelial growth medium (EGM-2 bullet kit; LONZA, NJ, USA) in standard culture conditions at 37 °C and 5% CO_2_. In vivo experiments were carried out using seven-week-old male Balb/c nude mice and Balb/c mice (Nara-Bio Company, Seoul, Korea). All animals were housed in specific pathogen-free conditions and maintained under the Institutional Animal Care and Use Committee (IACUC: 2021-0108A) at Hanyang University.

### Preparation of lactoferrin-glycyrrhizin conjugate (Lf-GL)

GL (50 × 10^–3^ M) was dissolved in distilled water and pH was adjusted using sodium carbonate (1.0 M) until the GL was fully dissolved into the solution. Next, sodium periodate (50 × 10^–3^ M) was dissolved in distilled water and the solution was kept in the dark. Then, the GL solution was slowly dropped into a SP solution to oxidize GL by stirring in the dark for 2 h at RT. Lf (0.0625 × 10^–3^ M) was dissolved in carbonate-bicarbonate buffer (CBS) (pH 10.5) at 4℃. The same volume of the Lf solution was added to the oxidized-GL solution in a drop-wise manner. The solution was reacted at 4℃ for 6 h. Finally, sodium cyanoborohydride solution (5 M, 10 μL) was added per milliliter. The reaction was allowed to proceed overnight at 4℃. The resulting solution was dialyzed with MWCO 50 kDa Centricon™ (Millipore, MA, USA) to remove unreacted substances. Then, it was lyophilized for 2 days to obtain the Lf-GL conjugate.

### Characterization of Lf-GL

Fourier transform infrared spectroscopy measurements (FT-IR, NICOLET IS50, Thermo Fisher Scientific) were conducted to identify the functional group of Lf-GL. The molecular weight of Lf-GL was determined by SDS-PAGE (12%-gel) and matrix-assisted laser desorption/ionization time-of-flight (MALDI TOF). MALDI-TOF was conducted at Seoul National University (Korea) using a MALDI-TOF Voyager DE-STR (Applied Biosystems, MA, USA). A sinapinic acid (Sigma-Aldrich) aqueous solution containing about 30% acetonitrile in 0.15% trifluoroacetic acid (Millipore) was used as a matrix. High performance liquid chromatography (Alliance HPLC e2695, Waters, MA, UK) using a gel permeation chromatography (GPC) column was performed to verify GL content in the Lf-GL conjugate. The mobile phase was composed of methanol, acetonitrile, water, and acetic acid in a ratio of 55:23.69:19.63:0.68. GL was dissolved in the mobile phase in different concentrations (2.5 to 200 × 10^–6^ M), and Lf-GL (25 × 10^–6^ M) was also dissolved in the mobile phase. Ultrahydrogel 120 Column (Waters) was used as a column, and the flow rate was 1 mL min^−1^. Then, absorbance was measured at 254 nm, and the results were calibrated with Empower software (Waters).

### Binding affinity against HMGB1

To confirm the binding affinity of Lf-GL to HMGB1, Surface Plasmon Resonance (SPR) was conducted at WoojungBSC (Korea). The Reichert SR7500DC system (Reichert technologies, NA, USA) and Scrubber2 software (Biologic software, FRA) were used, and a CMDH chip (Cat #: 13,206,066, Reichert technologies) was used for immobilization of the human recombinant HMGB1 protein (Abcam). HMGB1 protein was used as a ligand, and the analytes were HMGB1 antibody (Abcam), GL, Lf and Lf-GL conjugate. The immobilization buffer was S.A. (10 × 10^–3^ M, pH 4.5), and the running buffer was PBS (pH 7.4). The flow rate of the analyte was 30 μL min^−1^. The association and dissociation time were 3 min each, and measurements were conducted at room temperature. Results are expressed in response units (RU) over time.

### Cell viability test

Cell viability was determined using a cell counting kit-8 (CCK-8). U87MG and HUVECs (seeding density: 0.75 × 10^4^ cells per well) in 96-well culture plates were treated with GL, Lf, or Lf-GL (25 to 200 × 10^–6^ M of GL equivalent concentration) and were incubated for different periods of time (24, 48, and 72 h). After washing with PBS, they were treated with a cell culture medium containing a CCK-8 assay kit (Abbkine) and were incubated at 37 °C and 5% CO_2_ for 2 h. Then, absorbance was measured at 450 nm in a micro-plate reader (VLBL00D0, Thermo Fisher Scientific). Results were quantified using Sigma Plot (Systat Software, USA). To determine the effect of extracellular HMGB1 in GBM and endothelial cell proliferation, U87MG and HUVECs were seeded in 96-well culture plates (seeding density: 0.75 × 10^4^ cells per well) and GL, Lf, or Lf-GL (50 × 10^–6^ M GL equivalent concentration) were added to cells with or without HMGB1 protein (U87MG: 0.1 μg mL^−1^, and HUVEC: 1 μg mL^−1^). Cells were incubated for different periods of time (24, 48, and 72 h).

### Cell migration assay

A wound-healing scratch assay was performed in U87MG to evaluate the effect of GL, Lf and Lf-GL treatment on the cancer cell migration. Cells were seeded in 100 mm-cell culture dishes, 2.0 × 10^6^ cells per dish. After the cells were grown to 80% confluence, a scratch was made through the cell monolayer with a 200 μL pipet tip, and it was washed with PBS. Photographs (4 ×) were taken in each of three different scratch areas, and their location on the dish was marked. GL (50 × 10^–6^ M), Lf (5 × 10^–6^ M) or Lf-GL (1 × 10^–6^ M) dissolved in serum-free medium were added to cells with or without HMGB1 protein (0.1 μg mL^−1^). Then, cells were incubated for 24 h, and the same areas were re-photographed. Migration distances were examined using the scale bar.

### In vitro GBM cell uptakes of Lf-GL

To verify cellular uptake of Lf-GL, U87MG cells (seeding density: 2.5 × 10^4^ cells per well) in an 8 well Nunc™ Lab-Tek II chambered coverglass (Thermo Scientific) were treated with FITC-tagged Lf-GL (1 × 10^–6^ M) for different time periods (0 to 18 h). After washing with PBS, cells were treated with lysotracker (75 × 10^–9^ M) and incubated for 2 h. Then, cells were fixed with 4% paraformaldehyde followed by DAPI mounting. Confocal laser scanning microscopy (SP8 X, Leica, Germany) was used to observe intracellular fluorescence, and images were processed using LAS X software (Leica).

### HMGB1 ELISA

U87MG cells were seeded in 100 mm-cell culture dishes (2.2 × 10^6^ cells per dish) and treated with GL, Lf, or Lf-GL (50 × 10^–6^ M GL equivalent concentration) for 48 h. After incubating, cells were collected using a cell scrapper and were centrifuged for 3 min at 150 × *g*. Supernatant was collected as cell culture medium, and pellets were washed with cold PBS twice and transferred into a microcentrifuge tube. Tubes were centrifuged, and then pellets were resuspended in 500 μL 1X hypotonic buffer and incubated on ice. Then, 25 μL of NP40 was added and the mixture was agitated using a vortex mixer at the highest setting. Homogenates were centrifuged for 10 min at 842 × *g* at 4℃. Supernatant was collected as the cytoplasmic fraction, and pellets were resuspended in cell extraction buffer (50 μL) and were incubated for 30 min on ice with vortexing at 10 min intervals. Then, homogenates were centrifuged for 30 min at 14,000 × *g* at 4℃. Supernatant was collected as the nuclear fraction. The amount of HMGB1 in the samples was measured using a HMGB1 ELISA kit (Abbkine), and absorbance was measured at 450 nm in a micro-plate reader. A DNA assay (Quant-iT™ dsDNA Assay Kit, Invitrogen) was also conducted to express HMGB1 concentrations in equivalent amounts of DNA.

### Tube formation assay

A 24-well plate was coated with of Matrigel® (200 μL, Corning, NY, USA) and was incubated for 30 min in 37℃ incubators. HUVECs were seeded upon a coated Matrigel® 24-well plate (seeding density: 5.0 × 10^4^ cells per well). Subsequently, cells were treated with GL (50 × 10^–6^ M), Lf (5 × 10^–6^ M) or Lf-GL (1 × 10^–6^ M) and incubated for 24 h. Then, Calcein AM was added to each well, and the formation of tubes was observed using fluorescence microscopy. These tubes were quantified using Image J.

### Aorta ring assay

An aorta ring assay was conducted to confirm the anti-angiogenesis effect of Lf-GL ex vivo. A 48-well plate was coated with of Matrigel® (100 μL) and was incubated for 30 min or more in 37℃ incubators. In the meantime, thoracic aortas of 7-week-old male SD rats were excised. Then, the fat layer and outer membrane were removed, and rings approximately 1 mm in length were prepared. The prepared aortic rings were seeded on a coated Matrigel® of 48-well plate and were covered with Matrigel®. (100 μL) Subsequently, the aortic ring was treated with a control (with or without 25 ng mL^−1^ of VEGF) or different concentrations of Lf-GL complex. Medium was changed once every two days. Fourteen days after treatment, optical images of sprouting microvessels were obtained using microscopy magnification (× 40 or × 100). Sprouting microvessel area was quantified using Image J and Sigma Plot.

### Western blot

Total cellular protein concentration was determined using a BCA Protein Assay Kit (Pierce, Rockford, IL, USA). Equal amounts of protein from cell lysates were loaded onto a 12% sodium dodecyl sulfate–polyacrylamide gel. After electrophoresis, proteins were transferred to polyvinylidene fluoride membranes blocked with 5% bovine serum albumin for 1 h at room temperature and were diluted 1:500 with the indicated primary antibodies (rabbit anti-ERK1/2, anti-phospho-ERK1 and β-actin antibodies [Cell Signaling Technology, INC.]). Then, the membrane was washed 3 times for 15 min with tris-buffered saline containing Tween 20 and was incubated with HRP-conjugated secondary antibody for 1 h at room temperature. After washing three times for another 15 min with Tris-buffered saline with Tween 20, immune complexes were detected with an enriched chemiluminescent reagent (EZ-Western Lumi Femto, DoGen, South Korea) and staining was quantified by densitometry analysis using an Alpha Imager 2200.

### HUVEC co-cultured with U87MG

To evaluate the inhibitory effect of Lf-GL on neovascularization in the tumor microenvironment in vitro, U87MG cells were seeded in the Transwell® insert chambers that were 6.5 mm in diameter and had 0.4 μm pore sizes (seeding density: 2.5 × 10^4^ cells per well, Corning). HUVECs were seeded in the Transwell basal chamber (seeding density: 5.0 × 10^4^ cells per well). GL, Lf, or Lf-GL (100 × 10^–6^ M of GL equivalent concentration) were added to the insert chamber, and cells were incubated for 24 h. CCK-8 assay and HMGB1 ELISA were conducted at the basal chamber.

### In vivo pharmacokinetics study of Lf-GL

Balb/c mice were used for pharmacokinetics studies of GL and Lf-GL. Mice were administrated with GL equivalent concentration doses of 50 mg kg^−1^ body weight through tail vein injection. After the GL and Lf-GL injection, blood samples (500 μL) were collected by intra-cardiac puncture at time points of: 1 min, 3 min, 5 min, 10 min, 30 min, 1 h, 2 h, 2.5 h, 6 h, 12 h, 18 h, and 24 h. Blood samples were collected in ethylene-diamine-tetraacetic acid (EDTA) tubes (Microcollect, GSMEDITECH, Korea), and then centrifuged for 30 min at 842 × *g* in 4℃. Then, supernatants (50 μL) were mixed with methanol (100 μL). After vortexing for 10 min, extracts were centrifuged for 10 min at 10,000 × g. Thereafter, supernatants (100 μL) were mixed with the mobile phase solution (900 μL), which were then filtered with a syringe filter (0.22 μm). Samples were collected in 2 mL HPLC vials (Screw top vial, Waters) and analyzed with HPLC. The GL standard samples for calibration were also conducted using the same process. The absorbance of the samples was measured at 254 nm, and concentrations were calibrated through a GL standard curve.

### In vivo fluorescence tracer image of Lf-GL

For the fluorescence tracer images, Balb/c mice were administered either FITC-tagged GL or FITC-tagged Lf-GL at a GL equivalent concentration of 50 mg kg^−1^ body weight via tail vein injection. After 1, 10, 30, and 60 min, the experimental mice were sacrificed, and the organs were extracted. The fluorescence signals of FITC-tagged GL or FITC-tagged Lf-GL in organs were imaged using an in vivo imaging system (FOBI, CELLGENTEK, South Korea). The exposure time was fixed to 200 s for analyzing fluorescent signals from tissues. The quantified fluorescence signal of the brain at each time point was measured in terms of intensity units (IU, Intensity Min^−1^ Gain^−1^).

### In vivo analysis of GBM targeted-GL by HPLC in orthotopic GBM modeled mice brain

Orthotopic GBM mice model mice were used for quantification of GBM targeting efficacy. The brain tissue was homogenized in deionized water at a 33.3 w/v (%) using a FastPrep-24 5G (MP biomedical, South Korea). The GL were added to pooled brain homogenate to obtain seven-point calibration curves with a range of 10–1000 ng mL^−1^. Then, homogenate (50 μL) was mixed with methanol (100 μL). After vortexing for 10 min, extracts were centrifuged for 10 min at 10,000 × g. Thereafter, supernatants (100 μL) were mixed with mobile phase solution (900 μL), then filtered with a 0.22 μm syringe filter. Samples were collected in a 2 mL HPLC vial (Screw top vial, Waters, USA) and analyzed by HPLC at 254 nm. For the comparative study of GBM targeting, mice were administered GL and Lf-GL at a GL equivalent concentration dose of 50 mg kg^−1^ body weight through tail vein injection. Mice were sacrificed and brain tissues were collected 10 min after the GL and Lf-GL injection. The samples for quantification were also conducted using the same process as GL standard preparation. The absorbance of the samples was measured at 254 nm, and concentrations were calibrated suing the GL standard curve.

### In vivo systemic toxicity evaluation in GBM mouse model

To establish the orthotopic GBM mice model, GBM cells (U87MG, inoculation density: 1.0 × 10^6^ cells per 10 μL) were stereotactically injected into the right striatum of Balb/c nude mice. Then, mice were randomly divided into four groups (*n* = 5): control, GL, Lf, and Lf-GL treated group. From the day after tumor transplantation, the mice were administrated the drugs with GL equivalent concentration doses of 50 mg kg^−1^ body weight through tail vein injection every other day. Mice were observed for four weeks, and body weights and survival rates were recorded. After four weeks, mice were sacrificed, and organs were harvested and fixed with 4% paraformaldehyde for further analysis.

### In vivo histological study for Lf-GL efficacy on GBM mouse model

GBM mice were prepared and grouped as in previous experiments, but drugs were administrated every day (GL equivalent concentration dose of 50 mg kg^−1^ body weight through tail vein injection). After two weeks, mice were sacrificed, and the brains were harvested and fixed with paraformaldehyde (4%). Then, tissue processing was automatically conducted using a Leica TP1020 semi-enclosed Benchtop Tissue Processor (Leica Biosystems, Wetzlar, Hesse, Germany), followed by embedding in paraffin blocks. Paraffin blocks were sliced into 5 μm thick slices using a Leica RM2145 Microtome (Leica Biosystems). Nissl staining was used to stain brain tissue slides with pre-warmed 0.1% Cresyl Violet Stain Solution (Abcam). TUNEL assays were conducted following the manufacturer’s instructions. For immunofluorescence staining; anti-HMGB1 antibody (Abcam), anti-Ki67 antibody (Abcam), anti-CD31 antibody (Abcam), and anti-VEGF antibody (Abcam) were used as primary antibodies, and goat anti-rabbit IgG-H&L Alexa Fluor 488 and goat anti-rabbit IgG H&L Alexa Fluor 647 were used as secondary antibodies, followed by DAPI mounting.

### Statistics

All data are presented as the mean ± standard error of the mean (S.E.M). Statistical analysis was evaluated by Student’s t-tests or one-way analysis of variance (ANOVA) (Sigma Plot). *P*-values less than 0.05 are considered to be statistically significant.

## Results

### Synthesis and characterization of Lf-GL conjugate

To construct Lf-GL conjugate, we first oxidized GL using sodium periodate, a strong oxidant that is also used for opening glucose rings between vicinal diols [23], therefore aldehyde residues were exposed. Afterward, the aldehyde of oxidized-GL and amine of Lf undergo a Schiff-Base reaction to form a secondary amide bond. In this procedure, cyanoborohydride works as a reductant to reducing unstable secondary amide bonds to stable primary amide bond (Fig. 1b). The conjugation between GL and Lf was confirmed by FT-IR analysis, it exhibited amide I and amide II vibrations in both Lf and Lf-GL at 1,520 cm^−1^ and 1,630 cm^−1^ (Fig. S1) [24]. The observed peak at 1,035 cm^−1^ in both GL and Lf-GL indicates the primary alcohol stretch of GL. Therefore, the observation of the characteristic peaks of each Lf and GL in the Lf-GL conjugate suggests that the Lf-GL conjugation proceeded as intended. In the SDS-PAGE result, the band of Lf-GL shifted upward compared to native Lf due to the increased molecular weight by conjugation (Fig. S2). Moreover, in HPLC analysis, GL was detected at 17 min, while Lf-GL was detected at 3.5 min (Fig. S3). The faster elution time of Lf-GL compared to GL indicates that Lf-GL has a higher molecular weight than GL while maintaining sufficient GL content. MALDI-TOF was conducted to quantify the molecular weight of Lf-GL (Fig. S4). Average molecular weights of Lf and Lf-GL were 78,497.9 and 86,727.3 Da, respectively. Considering that the molecular weight of GL was 822.9 Da, the binding ratio between Lf and GL was 1:10.

### Lf-GL as a potent HMGB1 antagonist due to its high binding affinity to HMGB1

GL is one of the most potent inhibitors of HMGB1 and is known to block the nucleocytoplasmic translocation of HMGB1, thereby inhibiting its extracellular secretion and function as a DAMP in the tumor microenvironment [25, 26]. Therefore, we evaluated the respective binding affinities of different concentrations of GL, Lf, and Lf-GL to HMGB1 (Table 1 and Fig. S5). The K_D_ value of GL was 540.0 ± 20 μM for the HMGB1, whereas the values of Lf, Lf-GL (1:4), Lf-GL (1:10) and Lf-GL (1:15) were 165.0 ± 3, 595 ± 5, 23.1 ± 0.1 and 32.3 ± 0.2 nM, respectively. It is noteworthy that Lf and Lf-GL showed higher affinity for HMGB1 compared to the HMGB1 antibody (K_D_ value of 1.0 ± 3.0 μM) as well as GL, which is known as an antagonist to HMGB1 [13]. Among analytes, Lf-GL (1:10) showed the highest affinity with a K_D_ value of 23.1 ± 0.1 nM, whereas that of Lf-GL (1:15) containing more GL was 32.3 ± 0.2 nM. In general, the structural mechanism by which GL binds to HMGB1 is known as an extended hydrophobic interaction due to the formation of a concave surface formed by the two arms of HMG box A [27]. More specifically, the proximal glucuronic acid residue (attached to the terpenoid unit) of GL is key for the HMG interaction, in which hydrogen bonds and hydrophobic interactions stabilize the HMGB1-GL complexes. The bond stability is then further increased through intramolecular cysteine disulfide bonds. However, in our results, excessive amounts of GL (Lf-GL (1:15)) are shown to interfere with binding to HMGB1 by inducing intermolecular pi-pi stacking, hydrophobic interactions and hydrogen bonding [28]. In terms of solubility and activity, since pi-pi stacking occurs between the terpenoid unit structures of GL, conjugates with a reaction ratio of 1:10 or more show low solubility, and low solubility eventually leads to low activity. Taken together, Lf-GL (1:10) contains an adequate amount of GL without physical interference for HMGB1 binding, and the contribution of Lf to HMGB1 binding leads to improved affinity. Therefore, Lf-GL (1:10) was determined to exert optimal pharmacological properties and was subjected to further experiments.

**Table 1:**
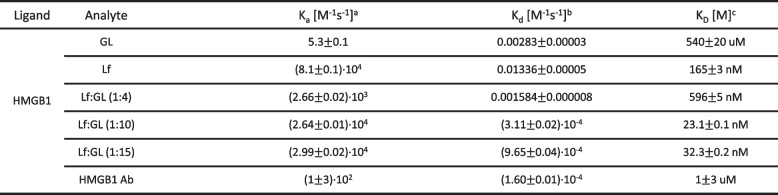
Binding affinity for HMGB1 according to the respective dissociation constants

^a)^Association rate constant

^b)^Dissociation rate constant

^c)^Equilibrium dissociation constant (K_D_ = K_d_ K_a_^−1^)

### In vitro, Attenuation of GBM proliferation by Lf-GL treatment

**Fig. 2:**
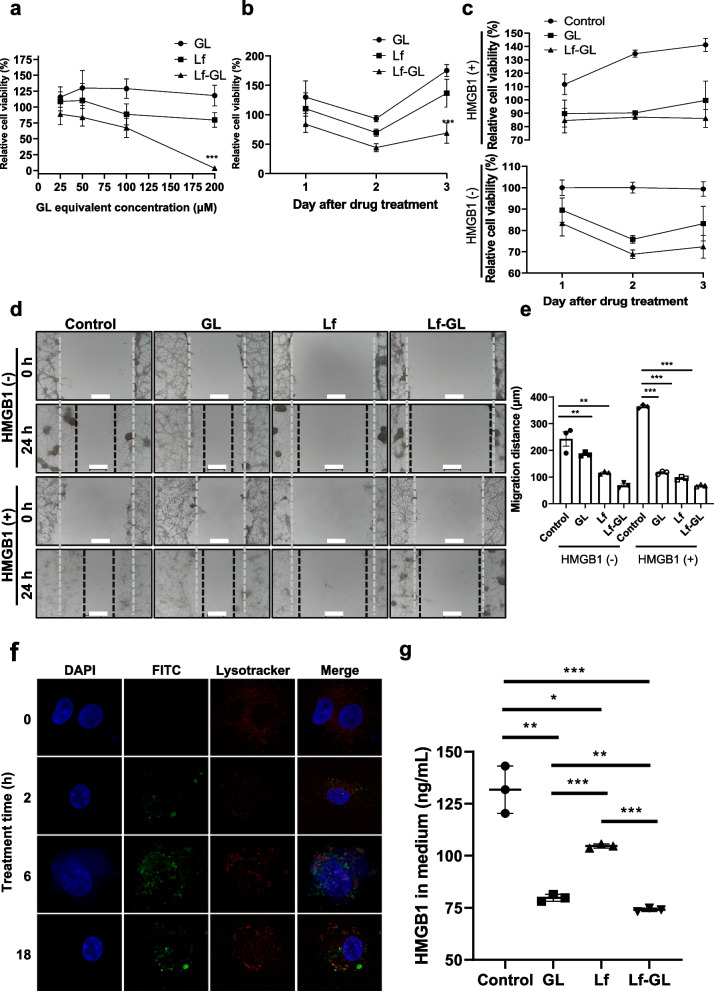
In vitro inhibitory effect of Lf-GL on GBM progression. **a** Relative viability of U87MG cells treated with GL equivalent concentrations of 25 μM to 200 μM, respectively, in GL-, Lf-, and Lf-GL- treated groups. Data are expressed as mean ± S.E.M (*n* = 6). ***P < 0.001 versus GL-treated group. **b** Monitoring tumor progression for 3 days in GL-, Lf-, and Lf-GL- treated U87MG cell. Data are expressed as mean ± S.E.M (*n* = 6). ***P < 0.001 versus GL-treated group. **c** Investigation of HMGB1-mediated GBM cell proliferation and its inhibitory ability of GL and Lf-GL for 3 days. Data are expressed as mean ± S.E.M (*n* = 8). **d** Wound healing assay to investigate HMGB1-mediated GBM cell migration and its inhibitory effect of GL, Lf, and Lf-GL. Data are expressed as mean ± S.E.M (*n* = 3). Red dashed lines indicate starting point. Blue dashed lines mark the location where the U87MG cells migrated over 24 h. Scale bar: 500 μm. **e** Inhibitory effect on tumor migration distance of Con-, GL-, Lf- and Lf-GL treatment groups according to the presence or absence of HMGB1. HMGB1 (-) indicates absence of HMGB1 treatment. HMGB1 ( +) indicates the presence of HMGB1 treatment. Data are expressed as mean ± S.E.M (*n* = 3). **P < 0.01 and ***P < 0.001. **f** Mechanism of cellular uptake of Lf-GL-FITC for 18 h by co-staining with DAPI and Lysotracker. **g** HMGB1 concentration in the medium of U87MG cells in different treatment groups. Data are expressed as mean ± S.E.M (*n* = 3). *P < 0.05, **P < 0.01, and ***P < 0.001Data are expressed as mean ± S.E.M (*n* = 3). *P < 0.05, **P < 0.01, and ***P < 0.001

A cell viability assay showed that Lf-GL more significantly reduced the number of GBM cells (U87MG) compared to Lf- and GL-treated groups at all GL equivalent concentrations of 25 μM to 200 μM (Fig. 2a and Fig. S6). Since the non-toxic concentration of Caco-2 cells considered normal cell was less than 100 μM while sufficient anti-tumor effect achieved above 25 μM (Fig. S7), further experiments were set at 50 μM. The inhibitory effects of GL, Lf and Lf-GL on cell growth were measured for 72 h (Fig. 2b). After 48 h treatment, GL, Lf and Lf-GL reduced the number of cells to 93.4 ± 4.8, 69.6 ± 5.7 and 44.1 ± 6.3% of the original count, respectively. Thereafter, cell number of the GL- and Lf-treated groups increased sharply over the next 24 h. In contrast, the Lf-GL-treated group efficiently attenuated the growth rate to 68.7 ± 15.9%. Considering the U87MG doubling time of 30.8 ± 2.5 h [29], it is anticipated that Lf-GL inhibits proliferation through cell division.

A major problem of chemotherapy is that high pressure in solid tumors reduces drug diffusion from blood vessels to tumor cells, resulting in poor penetration [30]. Therefore, anti-proliferative effect of Lf-GL on the three-dimensional (3D) structure of solid tumor-like glioma spheroids was investigated for 6 days (Fig. S8a). Morphological changes of spheroids including surface collapse or 3D structure destruction were not observed in the Lf-GL treatment. However, it sufficiently impeded the growth of tumor spheroids. The average volume of the control group increased gradually, which was 294% at day 6 compared to day 0 (Fig. S8b). In Lf-GL (1:4) and Lf-GL (1:15), spheroids increased in volume at day 6 by 185% and 179%, respectively, and moderate confinement effect was observed. Intriguingly, spheroids treated with the Lf-GL (1:10) increased by 156% at day 6, which was the slowest growth rate among other treatment groups. Furthermore, live/dead assay was conducted to determine the mechanism of Lf-GL in its tumor suppressive effect (Fig. S9). As a result, the growth of tumor spheroids was inhibited by anti-proliferation rather than by cell death such as apoptosis or necrosis. Consistently, the ratio of annexin V ( +)/PI ( +) cell population in the Lf-GL treatment group was 0.28%, which was lower than 1.40% in the control group (Fig. S10).

HMGB1-mediated GBM cell proliferation and its inhibitory effect of Lf-GL were investigated for 3 days. HMGB1 upregulated tumor growth by 110.7 ± 8.2, 133.4 ± 1.3 and 140.7 ± 5.2% on days 1, 2 and 3, respectively, compared to controls not treated with HMGB1. In this system, Lf-GL resulted the most robust inhibitory effect over 3 days (Fig. 2c). In the wound healing scratch assay, the HMGB1-treated cell (noted as HMGB1 ( +)) migrated 121.3 ± 42.8 μm more than the HMGB1-nontreated cell (noted as HMGB1 (-)) at 24 h (Fig. 2d). Similar to the above results, Lf-GL inhibited cell migration in the absence of HMGB1, but its effect was more dominant in the presence of HMGB1 (Fig. 2e). This could be the direct evidence that HMGB1 induces rapid tumor growth and metastasis, but Lf-GL can refrain HMGB1-mediated tumor progression with high affinity.

### Downregulation of cytoplasmic-HMGB1 level by LfR-mediated endocytosis of Lf-GL

The cellular uptake of Lf-GL was investigated to determine the cause of the lethal inhibitory effect on the proliferation of tumor cells. As a result, Lf-GL gradually internalized into U87MG cells up to 84.5% over 18 h (Fig. S11). To examine the endocytic mechanism of Lf-GL, the cellular uptake of FITC-tagged Lf-GL was monitored through flow cytometry in the presence of the Lf or Pitstop®, which is an inhibitor of clathrin-mediated endocytosis. After 2 h incubation, the cellular internalization of Lf-GL-FITC decreased about 17% in the presence of Lf, whereas it was not affected in the Pitstop® treatment. This result indicates that Lf-GL internalizes cells via the Lf receptor (LfR), but its endocytic pathway does not follow the clathrin-mediated pathway. Colocalization of Lysotracker and FITC-tagged Lf-GL was not fully identified in the overall images collected between 2 and 18 h, which corroborate the notion of clathrin-independent internalization of Lf-GL through LfR, a conclusion that is consistent with our previous studies (Fig. 2f) [16, 17, 31, 32]. Moreover, it was observed that even after 18 h of incubation, Lf-GL was remained in the cytoplasm, which indicates that it was protected from lysosomal degradation and expected to retain its functionality in inhibiting HMGB1. As a result, the concentration of HMGB1 released into the medium of U87MG cells was significantly lower in the Lf-GL treatment group (74.0 ± 0.9 ng mL-1) compared to the control, glycyrrhizic acid (GL), and lactoferrin (Lf) treatment groups, which had HMGB1 concentrations of 131.8 ± 11.4, 80.7 ± 4.5, and 114 ± 3.4 ng mL-1, respectively (Fig. 2g). This reduction in HMGB1 release was attributed to the ability of internalized Lf-GL to capture cytoplasmic HMGB1, which plays a critical role as a DAMP, and thereby inhibit its activity. The increased cellular uptake of Lf-GL facilitated this process and improved the efficiency of HMGB1 inhibition.

### Anti-angiogenic effect of Lf-GL

**Fig. 3:**
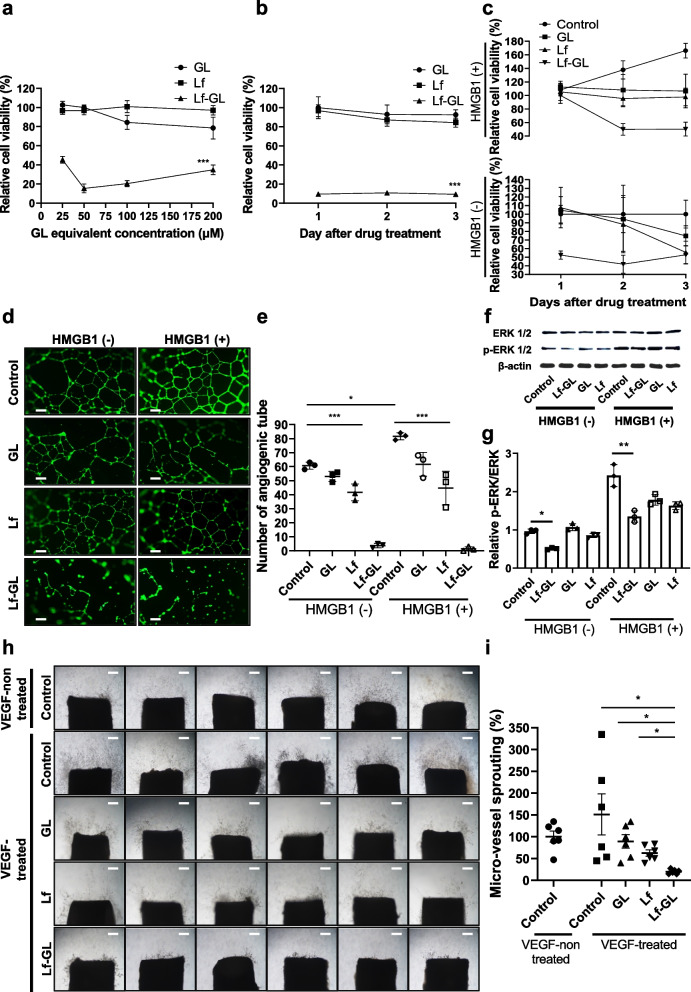
In vitro anti-angiogenic capacity of Lf-GL. **a** Relative viability of HUVEC cells treated with GL equivalent concentration of 25 μM to 200 μM, respectively, in GL-, Lf-, and Lf-GL- treated group. Data are expressed as mean ± S.E.M (*n* = 6). ***P < 0.001 versus GL-treated group. **b** Monitoring endothelial cell proliferation for 3 days in GL-, Lf-, and Lf-GL- treated HUVEC. Data are expressed as mean ± S.E.M (*n* = 6). ***P < 0.001 versus GL-treated group. **c** Investigation of HMGB1-mediated HUVEC proliferation and inhibitory ability of GL, Lf, and Lf-GL for 3 days. Data are expressed as mean ± S.E.M (*n* = 8). **d** Capillary tube formation assay to investigate HMGB1-mediated angiogenesis and its inhibitory effect of GL, Lf, and Lf-GL. Scale bar: 200 μm. **e** Quantification of tube formation in the absence or presence of HMGB1 in control, GL, Lf, and Lf-GL groups. HMGB1 (-) indicates absence of HMGB1 treatment. HMGB1 ( +) indicates the presence of HMGB1 treatment. Data are expressed as mean ± S.E.M (*n* = 3). *P < 0.05 and ***P < 0.001. **f** Western blot analysis for phospho- and total ERK levels in cell lysates. **g** Densitometric analysis of the related bands was expressed as the relative optical band density, which was corrected using total ERK proteins as a loading control and normalized against the untreated control. HMGB1 (-) indicates the absence of HMGB1 treatment. HMGB1 ( +) indicates the presence of HMGB1 treatment. Data are expressed as mean ± S.E.M (*n* = 3). *P < 0.05 and **P < 0.01. **h** Rat aortic ring assay to evaluate anti-angiogenic effect of GL, Lf, and Lf-GL under condition where endothelial cells, progenitor cells, and other angiogenic factors are interacting. Scale bar: 200 μm. **i** Quantification of micro-vessel sprouting. Data are expressed as mean ± S.E.M (*n* = 6). *P < 0.05

Angiogenesis is a fundamental event in solid tumor progression, where inhibition of neovascularization is considered a beneficial therapeutic approach. Therefore, we investigated the anti-angiogenic effects of Lf-GL on the neovascularization activities of endothelial cells. Cell viability assays showed that Lf-GL reduced the number of HUVEC the most (Fig. 3a, Fig. S12). Contrary to the mechanism in GBM cells, Lf-GL severely induced apoptosis and necrosis of endothelial cells (Fig. S13). Furthermore, the inhibitory effect of Lf-GL on endothelial cell proliferation, which is essential for angiogenesis, was monitored for 3 days. As a result, Lf-GL significantly inhibited endothelial cell growth compared to either the GL- treated or the Lf- treated groups for the entire time (Fig. 3b). To further investigate the contribution of extracellular HMGB1 to tumor angiogenesis, HMGB1 protein were treated to HUVEC to mimic the activity of HMGB1 in tumor microenvironment. HMGB1 indeed increased angiogenic potential of HUVEC by 108.2 ± 7.9, 137.9 ± 12.3 and 169.8 ± 18.1% on day 1, 2 and 3, respectively (Fig. 3c, viability comparison between control and HMGB1 ( +) control groups). Due to the high binding affinity of Lf-GL against HMGB1, it successfully attenuated the angiogenic effect triggered by HMGB1. It is known that HMGB1 triggers an angiogenic gene expression in endothelial cells and is involved in autocrine and paracrine cycle mechanisms resulting in positive enforcement of HMGB1 expression and that of its receptors like toll-like receptor 4 (TLR4) and RAGE [10, 33]. To this respect, Lf-GL may break this vicious cycle leading to endothelial cell sprouting and migration.

In the study of capillary tube formation, newly generated micro-vessels were increased by 21.0 ± 2.1% upon exposure to HMGB1 (Fig. 3d and e, comparison of angiogenic tube number between HMGB1 (-) control and HMGB1 ( +) control groups). In this regard, Lf-GL significantly decreased the tube formation in both absence and presence of HMGB1 treatment, respectively. The ERK and phosphorylated-ERK (p-ERK) signaling molecules, which are critically involved in endothelial cell proliferation, were investigated to gain further insight into the relationship between HMGB1 and HUVEC proliferation (Fig. 3f). After 24 h of treatment with HMGB1, the p-ERK level was significantly increased by 2.3 times compared to non-treatment of HMGB1 (Fig. 3g). However, Lf-GL reversed the ratio of p-ERK to ERK induced by HMGB1, inhibited most ERK phosphorylation, and showed substantial anti-angiogenic ability of Lf-GL.

Aortic rings obtained from rats were treated with GL, Lf and Lf-GL for 14 days to examine the anti-angiogenic effect under conditions where endothelial cells, progenitor cells, and other angiogenic factors are interacting (Fig. 3h) [34]. Vascular endothelial growth factor (VEGF) was used as a positive control group, and the outgrowth of endothelial tubules increased by 51.1 ± 105.2%. Furthermore, the vascular development from the intima/subintima of the aorta, sprouting from the initial vessels, and new micro-vessels formation was found in control (Fig. S14) On the other hand, Lf-GL decreased the sprouted area of the micro-vessel by 80.2 ± 4.5%, degenerated pre-existed blood vessels and suppressed new micro vascularization (Fig. 3i).

### Inhibition of tumor progression by Lf-GL in the tumor microenvironment

**Fig. 4:**
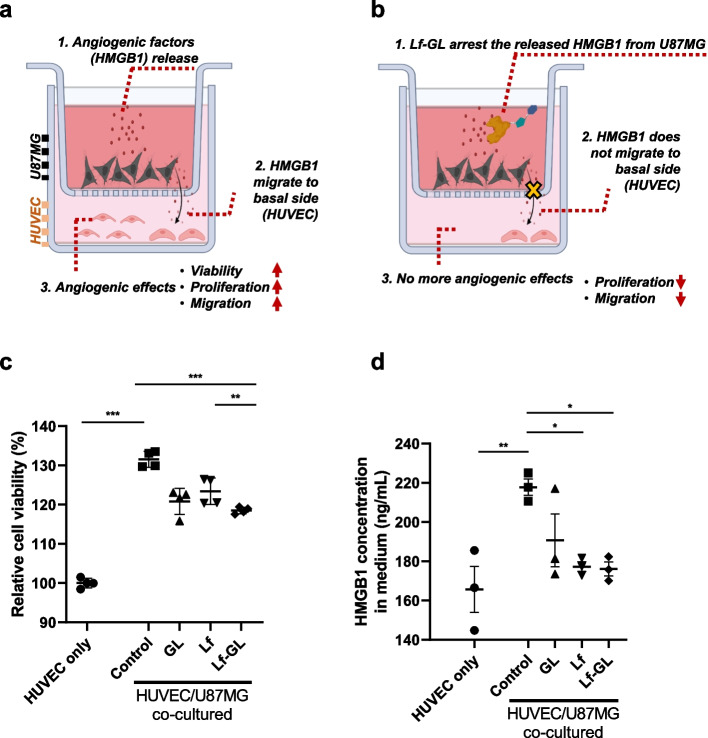
In vitro attenuation of GBM progression and tumor angiogenesis in the tumor microenvironment by Lf-GL modulating HMGB1 activity*.* **a** Schematic illustration of tumor microenvironment Transwell model in which HMGB1 released from U87MG affects the angiogenic properties of endothelial cells cultured in a basal chamber. **b** Schematic illustration in which Lf-GL effectively arrests tumor-released HMGB1 in the apical compartment and inactivates angiogenesis-related functions. The illustration was created with BioRender.com. **c** Quantification of HUVEC viability in the absence or presence of U87MG co-culture condition and the inhibitory effect of GL, Lf, and Lf-GL on HUVEC proliferation. Data are expressed as mean ± S.E.M (*n* = 4). **P < 0.01 and ***P < 0.001. **d** Quantification of HMGB1 concentration in the absence or presence of U87MG co-culture condition and the HMGB1 arresting effects of GL, Lf, and Lf-GL. Data are expressed as mean ± S.E.M (*n* = 4). *P < 0.05 and **P < 0.01

To clarify the influence of tumor released-HMGB1 on endothelial cell proliferation, we mimicked the tumor microenvironment with a co-culture system using 0.4 μm pore size trans-well plates. In this model, HUVECs were seeded in the basal chamber and U87MG cells were seeded in the apical compartment (Fig. 4a). As a result, the proliferation and viability of endothelial cells, which are essential for angiogenesis and microvascular sprouts, were increased by 31.6 ± 1.8% in the co-culture system (Fig. 4c). Accordingly, the HMGB1 concentration in the medium increased from 165.6 ± 16.6 to 217.8 ± 5.9 ng mL^−1^ in the co-culture condition (Fig. 4d, comparison between HUVEC only and control of HUVEC/U87MG co-cultured). This suggests that HMGB1 secreted from tumor is a key regulator for angiogenesis and that glioma and endothelial cells may have reciprocal effects through HMGB1. By arresting tumor secreted-HMGB1, GL, Lf and Lf-GL reduced endothelial cell proliferation by 10.7 ± 2.9, 8.2 ± 2.9 and 13.0 ± 0.7%, respectively (Fig. 4c), and HMGB1 level decreased to 190.7 ± 19.0, 177.3 ± 3.6 and 176.1 ± 4.9 ng mL^−1^, respectively (Fig. 4d). Furthermore, the results demonstrate that GL, Lf, and Lf-GL are able to reduce endothelial cell proliferation and HMGB1 secretion, although there were no significant differences among the treatment groups. It is possible that the lack of significant differences in the treatment groups may be due to the relatively short treatment duration or the limited sample size. Furthermore, the two bioactive molecules in the Lf-GL conjugate not only possess HMGB1 binding affinity but also have anti-cancer effects. Therefore, in this closed system, all the treatments were found to mitigate tumor-derived HMGB1 secretion, which has a reciprocal effect on angiogenesis. Based on the data, it can be inferred that GL, Lf, and Lf-GL have the potential to inhibit tumor angiogenesis by targeting HMGB1, which is known to be a crucial mediator of this process [12].

### Pharmacokinetics profiles and GBM targeting efficacy of Lf-GL

**Fig. 5:**
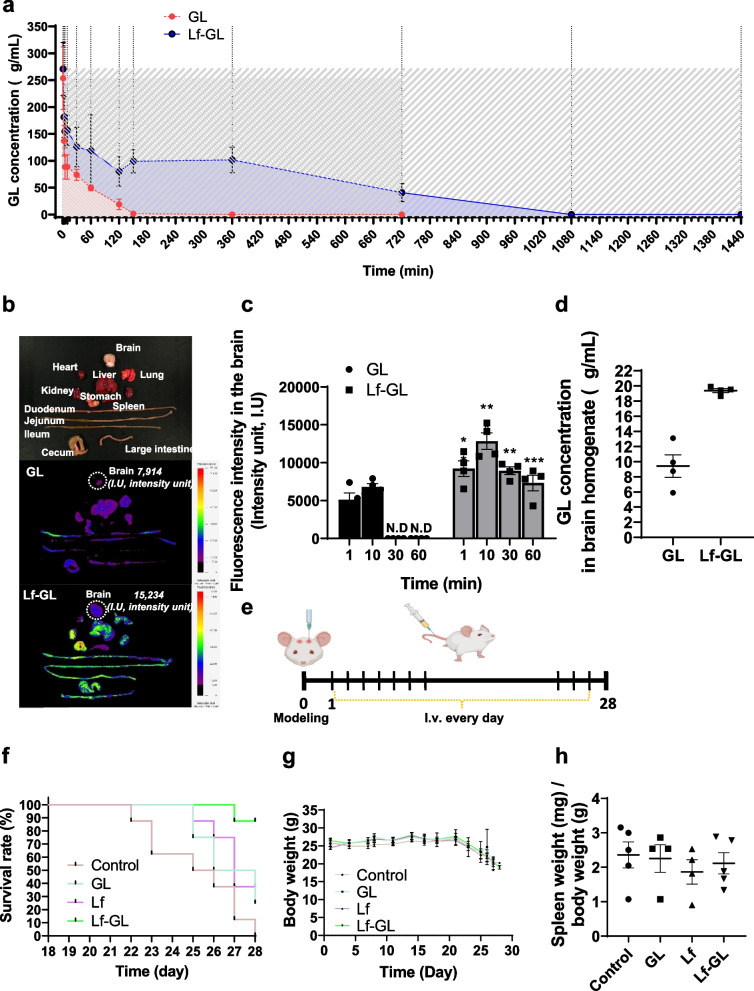
In vivo, the improved bioavailability of Lf-GL extended survival rate without systemic toxicity in orthotopic GBM mice model. **a** Pharmacokinetic study of GL and Lf-GL administered at a GL equivalent concentration of 50 mg kg.^−1^ investigated for 1440 min. Data are expressed as mean ± S.E.M (*n* = 8). **b** Fluorescence tracer images of GL and Lf-GL 10 min after intravenous injection in all organs. White dashed line indicates the detected fluorescence signal in the brain. **c** Quantification of fluorescence intensity at each time point in the brain of mice treated with GL or Lf-GL intravenously. Data are expressed as mean ± S.E.M (*n* = 4). **P < 0.01 and ***P < 0.001 versus GL at each time point. N.D: non-detectable. **d** GL concentration detected in brain homogenate 10 min after drug injection. Data are expressed as mean ± S.E.M (*n* = 4). **e** Schematic illustration of treatment plan. **f** Survival rate of PBS-vehicle- (Control), Lf-, GL-, and Lf-GL- treated group for 28 days. Data are expressed as mean ± S.E.M (*n* = 8). **g** Body weight transformation of PBS-vehicle- (Control), Lf-, GL-, and Lf-GL- treated group for 28 days. Data are expressed as mean ± S.E.M (*n* = 8). **h** Spleen weight (mg) / body weight of PBS-vehicle- (Control), Lf-, GL-, and Lf-GL- treated group after 28 days of treatment. Data are expressed as mean ± S.E.M (*n* = 5)

Pharmacokinetic studies of Lf-GL were carried out by administering a single dose (equivalent GL concentration dose of 50 mg kg^−1^ body weight) and were compared with free GL injected into healthy Balb/c mice intravenously. The plasma clearance of free GL and Lf-GL followed biexponential kinetics (Fig. 5a). Free GL was rapidly cleared from circulation 150 min after administration with a value of AUC_last_ of 6109.5 ± 3695.5 μgmin mL^−1^, and its half-life (T_1/2_) was approximately 3.8 ± 0.2 min. In contrast, Lf-GL remained high for longer periods (until 720 min), and its AUC_last_ increased by tenfold to 71,255.2 ± 45,319.5 μgmin mL^−1^ (Table 2).

**Table 2:**
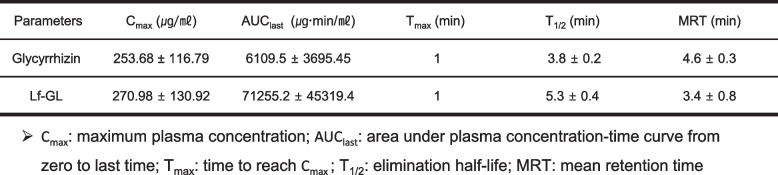
Pharmacokinetics parameters of intravenously administered glycyrrhizin and Lf-GL

To evaluate the GBM targeting efficacy of Lf-GL, the fluorescence of FITC-tagged-GL and FITC-tagged-Lf-GL was traced after intravenous injection (Fig. S15). As a result, the fluorescence intensities of both GL and Lf-GL were maximized at 10 min after injection and then gradually decreased after 1 h. However, due to the enhanced PK properties of GL by conjugating to Lf, the fluorescence intensity in the brain at 10 min increased from 7,914 to 15,234 intensity units (I.U.). Moreover, the fluorescence signal of Lf-GL in the brain was detected up to 60 min, whereas GL all disappeared after 10 min (Fig. 5b and c). In a study of the quantification of brain target-GL after 10 min injection, the GL concentrations detected in mice brain homogenates were 9.8 ± 2.3 and 19.3 ± 1.6 µg mL^−1^ in the GL and Lf-GL groups, respectively (Fig. 5d). This can be interpreted as the EPR effect, which is generally arise by abnormal angiogenesis and tumor pressure in GBM [35, 36]. However, the amount of GL that permeated the BBB and accumulated in GBM was significantly higher for Lf-GL, demonstrating that GBM targeting depends on the interaction between Lf and LfR expressed in BBB and GBM.

### Extension of survival rate with minimal systemic toxicity through Lf-GL treatment

Survival and systemic toxicity studies were performed by intravenous administration of daily doses (equivalent GL concentration dose of 50 mg kg^−1^ and Lf concentration dose of 5 mg kg^−1^) to GBM-mice model for 28 days (Fig. 5e). As a result, PBS vehicle-administered (Control) began to die on day 21 and none survived until day 25. The GL had a modest effect on long-term survival in that survived 4 more days. Interestingly, the Lf or Lf-GL-treated mice had an additional 5 days in the overall survival rate. Moreover, 25% and 87.5% of the mice in the Lf-and Lf-GL-treated groups survived up to 28 days, respectively (Fig. 5f). Weight loss an important parameter in repeated-dose toxicology studies [37], were measured throughout the experiment to investigate the systemic toxicity of Lf-GL upon long-term repeated dosing (daily administered for 28 days) (Fig. 5g). There was no difference in body weight in all experimental groups until day 21, the time of death due to tumor development. The spleen weight/body weight ratio was evaluated to investigate whether spleen enlargement occurred due to unwanted immune stimulation after repeated administration. As expected, there was no significant difference between groups (Fig. 5h). Histopathological analyses of the heart, liver, lung, spleen, and kidneys were conducted and there was no detection of immune responses leading to histological lesions composed of inflammatory cells such as neutrophils, macrophages, and B lymphocytes in all groups (Fig. S16). Therefore, we inferred that repeated administration of Lf-GL itself does not cause an unnecessary immune response to the reticuloendothelial system (RES) and has no systemic toxicity. Furthermore, the pharmacokinetic (PK) properties of Lf-GL were improved compared to free-GL (as shown in Table 2), which led us to anticipate that Lf-GL would be a suitable formulation to enhance tumor targeting by increasing bioavailability. In contrast, free-GL is rapidly eliminated from the body without being effectively metabolized [38], which may limit its efficacy as a therapeutic agent for tumor treatment.

### Reduction of biomarkers related to tumor development by inhibition of HMGB1 activity

**Fig. 6:**
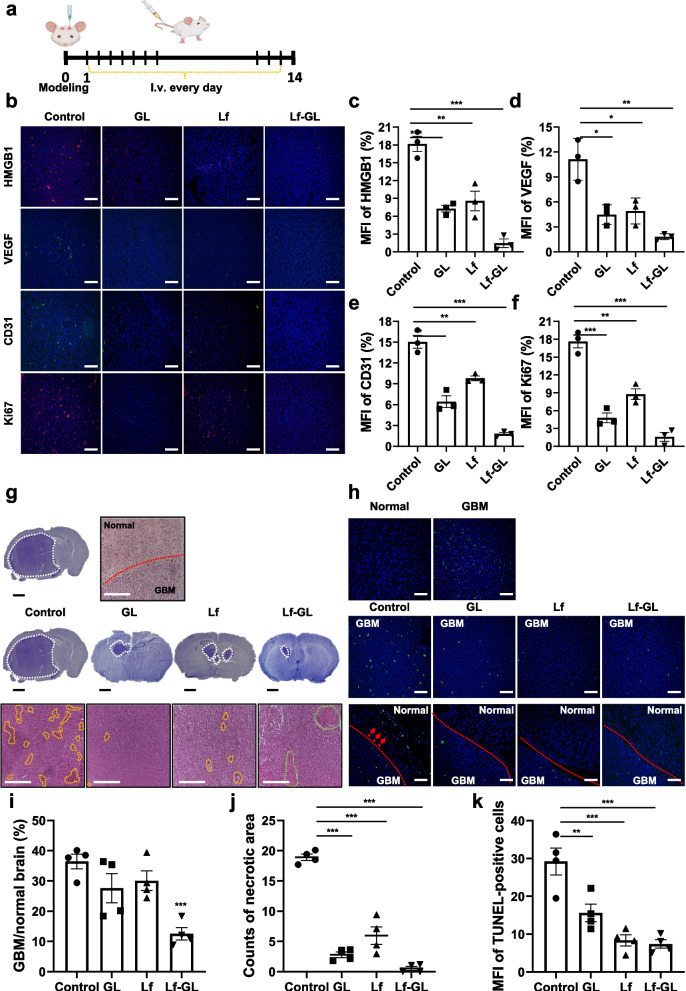
In vivo effective antitumor and anti-angiogenesis of Lf-GL in orthotopic GBM mouse model. **a** Schematic illustration of treatment plan. **b** Immunofluorescence staining of HMGB1, VEGF, CD31, and Ki67 in GBM tissues after 14 days of treatment. Scale bar: 100 μm. **c** Quantification of HMGB1 mean fluorescence intensity (MFI). Data are expressed as mean ± S.E.M (*n* = 3). **P < 0.01 and ***P < 0.001. **d** Quantification of VEGF mean fluorescence intensity (MFI). Data are expressed as mean ± S.E.M (*n* = 3). *P < 0.05 and **P < 0.01. **e** Quantification of CD31 mean fluorescence intensity (MFI). Data are expressed as mean ± S.E.M (*n* = 3). **P < 0.01 and ***P < 0.001. **f** Quantification of Ki67 mean fluorescence intensity (MFI). Data are expressed as mean ± S.E.M (*n* = 3) **P < 0.01 and ***P < 0.001. **g** Nissl and H&E staining. White dashed lines in Nissl staining represents the GBM area of the whole brain. Red dashed line in H&E staining represents the boundary between the tumor and normal regions. Scale bar: 100 μm. **h** TUNEL assay of PBS-vehicle- (Control), Lf-, GL-, and Lf-GL-treated group. Red dashed line represents the boundary between the tumor and normal regions. Red arrows indicate tumor infiltration into surrounding tissues. Scale bar: 100 μm. **i** Proportion of GBM in the brain after treatment. Data are expressed as mean ± S.E.M (*n* = 4). ***P < 0.001 versus Control. **j** Counts of necrotic areas on the H&E staining. Data are expressed as mean ± S.E.M (*n* = 4). ***P < 0.001. **k** Quantification of TUNEL mean fluorescence intensity (MFI). Data are expressed as mean ± S.E.M (*n* = 4). **P < 0.01 and ***P < 0.001

The restriction effect of Lf-GL on tumor development was assessed with intravenous administration of daily doses (equivalent GL concentration dose of 50 mg kg^−1^ and Lf concentration dose of 5 mg kg^−1^) up to 14 days after GBM modeling (Fig. 6a). The mean fluorescence intensity (MFI) of HMGB1 was highest in the PBS vehicle-administered (control) group, with a value of 15.3 ± 1.7. In contrast, the GL-, Lf-, and Lf-GL- administration group reduced the MFI of HMGB1 to 6.2 ± 0.8, 7.9 ± 1.4, and 1.7 ± 1.0, respectively, and the distribution area was relatively narrow (HMGB1 immunofluorescence staining in Fig. 6b and c). This could be interpreted as Lf-GL arrests the tumor-released HMGB1 at an early stage and prevents aggressively worsening tumor growth. Furthermore, HMGB1 promotes vascular endothelial growth factor (VEGF) secretion in a RAGE-dependent manner and is also known to stimulate endothelial progenitor cells homing to tumor tissue [39]. Therefore, the control group, which had a high MFI value of HMGB1, also showed the highest MFI value of VEGF among the experimental groups but it gradually decreased by the treatments (VEGF immunofluorescence staining in Fig. 6b and d). GBM is a tumor in which angiogenesis actively occurs, although a marked imbalance between angiogenesis-promoting factors and anti-angiogenic factors in the tumor microenvironment results in abnormal blood vessels. To this end, tumor vascularization of CD31, an endothelial cell marker, was investigated. Expression of CD31 associated with abnormal blood vessels was detected high in the control group with an MFI value of 14.7 ± 1.4. Moreover, its CD31-positive blood vessels dispersed throughout the GBM lesions. In contrast, the MFIs of CD31 in the GL-, Lf-, and Lf-GL- administered group decreased by approximately 4-, 2-, and sevenfold, respectively (CD31 immunofluorescence staining in Fig. 6b and e). Consistent with the CD31 staining results, tumor cell proliferation marker Ki67 was also expressed abundantly in the control group, but treatments decreased similarly to CD31(Ki67 immunofluorescence staining in Fig. 6b and f).

### Significant GBM therapeutic effect by Lf-GL treatment in orthotopic GBM mouse model

The GBM/normal brain ratio was determined through Nissl staining. GL-, Lf-, and Lf-GL-administered group reduced the ratio to 26.4 ± 5.7, 29.7 ± 3.3, and 8.9 ± 3.2, respectively. On the other hand, the PBS vehicle-administered group (Control) showed the highest ratio of 37.5 ± 2.3 (Fig. 6g and i, Nissl staining). In the H&E results, the tumor tissue had a dense structure and consisted of polymorphic cells with oval nuclei 10 to 20 μm in diameter. A large number of necrotic areas were observed in the GBM region of the control groups, whereas it significantly decreased in the GL-, Lf-, and Lf-GL-administered groups (Fig. 6g and j, H&E staining). It was noteworthy that hemorrhage and thrombosis were observed in the GBM region of the Lf-GL-administered group (Fig. 6g, green dashed line in Lf-GL group). This is because Lf-GL limits tumor vascularization through apoptosis in endothelial cells, leading to oxygen and nutrient deprivation.

TUNEL assays are widely used for the detection of apoptotic events by terminal deoxynucleotidyl transferase (TdT), which labels blunt ends of double-stranded DNA fragments. Therefore, a TUNEL-positive signal should not be found in the GBM region because the anti-apoptotic mechanism is abnormally upregulated in tumors, which enables cancer cells to avoid apoptosis [40]. However, over the past decade, numerous shortcomings have been pointed out in the evaluation of apoptosis through the TUNEL assay [41]. Many reports claim that TUNEL staining is non-specific in that it labels all free 3'-hydroxyl ends, irrespective of the molecular mechanism designed as planned. For this reason, it also labels non-apoptotic cells such as necrotic degenerative cells [42]. Therefore, the morphology should be assessed concurrently when performing the TUNEL assay to differentiate between apoptotic cells and necrotic cells. Consistent to recent claims, TUNEL-positive cells were predominant in the innermost GBM region where a large number of necrotic cells were observed in the H&E results. On the other hands, the TUNEL signal was not detected in the normal brain regions (Fig. 6h). In this respect, TUNEL-positive cells can be considered as necrotic tumor cells that play a catalytic role in tumor progression and aggressiveness. As results, the PBS-vehicle administered group (control) had the most TUNEL-positive cells among the experimental groups with an MFI value of 28.5 ± 4.8. Moreover, those TUNEL-positive cells invaded normal areas and metastasized to surrounding tissues. Meanwhile, the MFI of GL-, Lf-, and Lf-GL- administration group decreased to 16.3 ± 2.8, 9.4 ± 1.6, and 9.1 ± 0.9, respectively, (Fig. 6h and k) and most of them were confined to the tumor site without migration to the normal region (Fig. 6h, images of tumors and normal areas separated by red dashed line).

## Discussion

In this study, we demonstrated that the extracellular HMGB1 protein, recognized as a DAMP in the tumor microenvironment, plays a critical role in tumor angiogenesis and development. The HMGB1 released from the necrotic tumor executes alarmin functions, activating tumor growth signaling via binding to a variety of receptors including TLR2/4, RAGE, and C-X-C motif chemokine receptor 4 (CXCR-4) [43, 44]. Moreover, it is known that tumor cells secrete HMGB1 to recruit endothelial cells and facilitate a tumor vascular system by the crosstalk [45, 46]. To this respect, a few small molecules such as GL, acetylcholine, and GTS-21 (also known as DMBX-A) have been characterized as HMGB1 inhibitors, binding directly to inactivate its function or expression. GL is known to be a potent inhibitor of HMGB1, which of the equilibrium dissociation constant (K_D_) value was previously reported to be 170 ± 3 μM for box A and 140 ± 3 μM for box B [13]. However, a small molecule, GL, suffers from poor pharmacokinetics (PK) and pharmacodynamic (PD) properties, resulting in deficient delivery to the site-of-action.

To address this unmet clinical application, we established a novel Lf-GL conjugate with a ligation ratio of 1:10 (Fig. 1b). Notably, we found for the first time that Lf has high affinity for HMGB1 with a K_D_ value of 165.0 ± 3 nM, which is approximately ~ 10 folds higher than that of HMGB1 antibody (K_D_ value of 1.0 ± 3.0 μM) (Table 1). In the case of protein–protein interactions (PPIs), the combination of hydrophobic interactions, van der Waals forces, and metal or ion bridges at specific binding domains on each protein are all significant factors. These domains can be small binding clefts or large surfaces of the protein. In this context, Lf is an iron-binding protein consisting of two connected lobes, each with a binding site located in a cleft. Assuming the binding of Lf to the HMGB1 protein, it is expected that Lf, a globular glycoprotein with a molecular mass of about 80 kDa, would participate in many non-covalent interactions with HMGB1. Thereafter, the two arm-structure of HMGB1 and each lobe of Lf undergo a conformation change to become a stable complex. It is a valuable finding that Lf exhibits significant affinity for HMGB1, but more structural information of this complex must be further investigated by specific analysis such as NMR spectroscopy and thermodynamic characterization. Taken together, the exceptional contribution of Lf to HMGB1 binding resulted in a significant affinity with a K_D_ value of 23.1 ± 0.1 nM when conjugated between Lf and GL at a ratio of 1:10.

The observation that Lf-GL treatment shows some cytotoxicity to cancer cells in the absence of HMGB1 is an interesting finding that highlights the potential complexity of the Lf-GL complex's mechanism of action. While the cytotoxic effects of Lf-GL were only observed at relatively high concentrations, it is still important to understand the underlying mechanism of this effect, as it may have implications for the safety and efficacy of Lf-GL as a therapeutic agent. The observed cytotoxicity may reflect the unique properties of the Lf-GL complex, which is composed of two bioactive molecules with diverse mechanisms of action. Lactoferrin, for example, has been shown to interact with a variety of cell surface receptors and signaling pathways that modulate cell proliferation, differentiation, and apoptosis [47]. Similarly, glycyrrhizin has been shown to interact with a variety of signaling pathways, including the NF-κB pathway, which plays a critical role in regulating cell survival and apoptosis [48]. However, further research is needed to fully understand the mechanism underlying the observed cytotoxic effects of Lf-GL treatment. It is possible that Lf-GL interacts with additional signaling pathways or receptors in a way that induces cytotoxic effects, or that the observed cytotoxicity is due to the accumulation of Lf-GL in cancer cells. Moreover, it is important to determine whether the observed cytotoxicity is clinically relevant, as the concentration of Lf-GL required to induce these effects may be difficult to achieve in vivo. Overall, the observation of cytotoxicity in the absence of HMGB1 highlights the need for further investigation into the unique properties and mechanisms of action of the Lf-GL complex. While the primary focus of this study is on the anti-angiogenic effects of Lf-GL, these additional observations underscore the potential complexity of this therapeutic approach, and the need for further preclinical and clinical studies to fully understand its safety and efficacy.

Behalf of the high binding affinity to HMGB1, Lf-GL (1:10) exerted dominant antitumor activity in both 2D and 3D tumor cell spheroid culture system (Fig. 2d and e). Tumor cell migration and invasion, a critical factor in tumor progression and metastasis, were effectively attenuated in the presence of HMGB1 (Fig. 2). It is important to arrest HMGB1 in terms of tumor malignancy because extracellular HMGB1 is known to bind RAGE, which is overexpressed in high grade tumors [49]. Once RAGE is engaged by HMGB1, several signaling pathways such as MAPK and NF-_K_B become activated, thereby reprogramming cellular properties and progress to tumor migration and proliferation. In our study, Lf-GL arrested HMGB1 not only in the extracellular but also in the cytoplasmic fraction (Fig. 2g). This was attributed to the interaction between Lf and LfR that expressed on GBM cells, and we further explored the endocytosis mechanisms. As results, Lf-GL sufficiently internalized GBM cells (84.5%) via Lf receptor (LfR), but its endocytic pathway was not dependent on the clathrin-mediated pathway (Fig. 2f). The mechanism of internalization of Lf through GBM cell remains controversial. There is evidence that internalization of nanoparticles functionalized with Lf is partially (40%) decreased by inhibitors of clathrin-mediated endocytosis of mannitol (200 mM) [20], which is not consistent with our result. However, the endocytic pathway also depends on the physical, chemical, and geometrical properties of the molecules. Studies have shown that Lf-bindable receptors include such as CD14 [50], LDL receptor-related protein-1 (LRP-1/CD91) [51], intelectin-1 (omentin-1) [52], toll-like receptor 4 (TLR4) [53], cytokine receptor 4 (CXCR4) [54], as well as heparan sulfate proteoglycans (HSPGs) [55]. These HSPGs are macromolecules that exist on the cell surface and extracellular matrix, consisting of a core protein decorated with covalently linked glycosaminoglycan (GAG) chains. As Lf can bind to a range of multiple receptors, it presents a challenge in determining whether Lf undergoes endocytosis through a clathrin-dependent pathway. Therefore, it can be concluded that the unique properties of Lf-GL may not heavily rely on the clathrin-mediated endocytic pathway. Due to the independence of clathrin-mediated endocytic pathway which can circumvent lysosomal protein degradation [56], Lf-GL was capable to arrest HMGB1 in the cytoplasm of GBM cell. Arresting HMGB1 in the cytoplasmic fraction is regarded as significant because the function of HMGB1 is dominated by its subcellular location. In general, HMGB1 is localized in the nucleus, which regulates DNA stability and repair. However, the translocated HMGB1 from the nucleus governs the tumor cell proliferation, invasion, and metastasis [57]. It has been reported that the translocation of HMGB1 from the nucleus to the cytoplasm actively promotes autocrine, which is governed by post-translational modification such as phosphorylation [58]. Then, phosphorylated HMGB1 is transported to the cytoplasm and subsequently secreted out of the cells, where it plays a pivotal role in tumor progression [59, 60]. Therefore, we hypothesized that enhanced cellular uptake of Lf-GL provided more benefit in arresting nuclear-to-cytoplasmic translocated-HMGB1 as an important factor in the disturbance of tumor cell proliferation compared with other treatment groups.

In the tumor angiogenic environment, HMGB1 is known to act as a proinflammatory cytokine that stimulates the expression of vascular endothelial growth factor (VEGF) and triggers migration and sprouting of endothelial cells [61]. Multiple sources of HMGB1 exist in the tumor microenvironment; 1) necrotic tumor cells passively release HMGB1 into the peripheral space; 2) tumor-associated macrophages (TAMs) actively secrete HMGB1 and other vascularization growth factor [62]; 3) it also has been shown that endothelial cells secrete HMGB1 after activation and re-activate themselves upon exposure to that of previously secreted HMGB1 [61]. To this end, we constructed tumor microenvironment-mimic model to investigate the restriction effect of Lf-GL on HMGB1, which plays a critical role in crosstalk between tumor and endothelial cells (Fig. 4). As results, Lf-GL arrested HMGB1 secreted by GBM cells, attenuating angiogenic functions and inhibiting migration to endothelial cells. Therefore, the proliferation and motility of endothelial cells were efficiently inhibited in the tumor microenvironment (Fig. 4b). Taken together, Lf-GL efficiently targeted the GBM through interactions with LfR, inhibited GBM cell proliferation and growth, and arrested HMGB1, which is considered as a key factor for tumor growth and angiogenesis. Therefore, in the tumor microenvironment, Lf-GL appears to be a potent anti-tumor and anti-angiogenesis therapeutic agent.

GL, a small molecule used for anticancer treatment, often suffers from poor pharmacokinetics (PK) and pharmacodynamic (PD) properties due to its small size. Small molecules typically exhibit short half-lives and broad distributions, making them difficult to target at the site of action for long periods of time [63]. In addition, the small size of the pharmacophore often results in non-specific binding, resulting in narrow therapeutic indices. In this regard, protein conjugates are generally reported as a strategy for improving the PK-PD properties of small molecules, which may significantly increase their therapeutic potential. Targeted protein conjugates, developed primarily from antibodies, antibody derivatives, or endogenous proteins, are designed to improve delivery to the site of action and thereby enhance the therapeutic index of the agent [64]. Our results suggest that the conjugation of Lf, an autocrine endogenous protein, to GL significantly improved the PK properties of the molecules and increased its delivery to GBM (Table 2 and Fig. 5a-d). The enhanced PK properties of Lf-GL enabled targeting of LfR expressed in BBB and GBM cells, and the accumulated amount was twofold higher than GL without Lf conjugation (Fig. 5b-d).

Clinically, the development of necrotic cores in cancer patients is correlated with high-grade disease, increased tumor size, and poor prognosis [65]. More specifically, tumor necrosis, a common occurrence in malignant brain tumors, begins with cell swelling and releases cytoplasmic contents such as HMGB1 into the extracellular space through plasma membrane rupture. These released molecules promote tumor progression by recruiting immune cells that can trigger an inflammatory response, increasing the possibility of epigenetic changes or proto-oncogenic mutations, and inducing angiogenesis, cancer cell proliferation and invasion. In this respect, the released HMGB1 plays a critical role in regulating the epithelial mesenchymal transition (EMT), which initiates tumor invasion and metastasis [66]. Consistent to clinical view, in our study, necrotic tumor cells sustainedly secreted HMGB1 and triggered cascade response, leading to persistent necrosis of the lesion. Moreover, HMGB1 released into the extracellular environment infiltrates into the surrounding brain tissue to proliferate the surrounding tumor cells, causing the regeneration of small blood vessels to promote spreading and tumor growth [67]. At this point, Lf-GL, which has a high binding affinity for HMGB1, eliminate the activity involved in tumor development and metastasis by arresting HMGB1. Thus, it prevents infiltration of tumor cells into surrounding tissues, which occurs through tumor necrosis.

As mentioned in the manuscript, HMGB1 is a pro-tumorigenic factor that can promote tumor growth, angiogenesis, and inflammation. However, recent studies have also shown that early DAMP signals such as HMGB1 can play a role in recruiting tumor infiltrating lymphocytes (TIL), which can be beneficial for additional immunotherapy [68]. This raises the question of whether Lf-GL treatment could reduce the population of TIL in the tumor microenvironment. Nevertheless, lactoferrin has been shown to enhance the immune response by promoting the activation and proliferation of immune cells, including T cells and natural killer cells [69]. This immune-enhancing effect may counteract the downregulation of TILs caused by anti-angiogenic therapy. Consequently, Lf-GL has the potential to mitigate immunosuppression resulting from HMGB1 downregulation in the tumor microenvironment. However, further research is required to fully comprehend lactoferrin's role in addressing TIL downregulation and its complementarity with anti-angiogenic therapy. Secondly, combining anti-angiogenic therapy with other treatments such as immunotherapy or immune checkpoint blockade may help mitigate concerns related to immunosuppression and may increase TIL infiltration [70]. Recent studies have shown promising results with combination therapies in preclinical models of GBM, suggesting that such approaches may be a promising avenue for future research [71]. However, further investigation is needed to determine the optimal combination therapies for treating GBM and to fully understand the effects of Lf-GL treatment on TIL populations in the tumor microenvironment. Overall, combination therapies hold great promise for improving the efficacy and safety of anti-angiogenic therapy in GBM and other cancers and may provide a path forward for addressing some of the key challenges facing cancer treatment today.

## Conclusion

In this study, we developed Lf-GL with a high binding affinity for HMGB1 that acts as a tumor-promoting cytokine and is involved in angiogenesis and tumor progression in the tumor microenvironment. The binding affinity of Lf-GL to HMGB1 was 45-fold higher than that of the HMGB1 antibody. Lf of the Lf-GL improved targeting to GBM through interaction with LfR, allowing Lf-GL to arrest released HMGB1 present in the extracellular space as well as in the cytoplasmic fraction. Therefore, Lf-GL effectively inhibited the cascade (tumor angiogenesis, tumor progression, infiltration into surrounding tissues, etc.) induced by HMGB1 secreted from necrotic tumor. Furthermore, Lf addressed the unfavorable PK property of GL, delivering it to the site of action. Thus, in the orthotopic GBM mice model, Lf-GL significantly prolonged the survival period and reduced the tumor volume by 32% compared to the untreated group. Consequently, our findings suggest a new HMGB1 inhibition approach to comprehensive GBM treatment and may offer a novel platform for the treatment of HMGB1-related disorder.

## Supplementary Information

**Additional file 1: Figure S1.** FT-IR spectra of GL, Lf and Lf-GL respectively. The conjugation between GL and Lf was confirmed by Fourier transform infraredanalysis. The amide I and amide II vibrations observed at 1,523 cm^-1^ and 1,630 cm^-1^ in both Lf and Lf-GL. The 1,035 cm^-1^ indicates primary alcohol stretch of GL. **Figure S2.** SDS-PAGE result of Lf and Lf-GL. The successful synthesis of Lf-GL could be assumed from the sodium dodecyl sulfate polyacrylamide gel electrophoresisresults as a molecular weightrise in the band above 80 kDa, the MW of native Lf. **Figure S3.** HPLC result of GL and Lf-GL. High performance liquid chromatographyusing gel permeation chromatographycolumn was performed to verify GL content in Lf-GL conjugate. Mobile phase is composed of methanol, acetonitrile, water, and acetic acid in a ratio of 55:23.69:19.63:0.68. GL was dissolved in mobile phase in different concentrationand Lf-GLwas also dissolved in mobile phase. Ultrahydrogel 120 Columnwas used as column, flow rate was 1 mL min^-1^. Then, absorbance was measured in 254 nm and results were calibrated with Empower software. **Figure S4.** MALDI-TOF result of Lf and Lf-GL. MALDI-TOF was conducted at Seoul National Universityusing MALDI-TOF Voyager DE-STRand sinapinic acidaqueous solution containing about 30% acetonitrile in 0.15% trifluoroacetic acidwas used as a matrix. Average molecular weight of Lf-GL is 86,727.3 ± 98.1 Da. Considering the molecular weight of Lf is 78,497.9 Da and GL is 822.9 Da, binding ratio of Lf:GL is 1:9.6.** Figure S5.** Binding affinity against HMGB1. Surface Plasmon Resonancewas conducted at WoojungBSC. The Reichert SR7500DC systemand Scrubber2 softwarewere used and the CMDH chipwas used for immobilization of the human recombinant HMGB1 protein. HMGB1 protein was used as ligand and analytes were HMGB1 antibody, GL, Lf and Lf-GL conjugate. Immobilization buffer was 10 mM S.A.and running buffer was 1× PBS. The flow rate of the analyte was 30 μL min^-1^. The association and dissociation time were 3 min each and conducted at room temperature. Results are expressed in response unitsover time. **Figure S6.** Live and dead assay of U87MG in the Control, GL, Lf, and Lf-GL group. The cells were treated with GL equivalent concentration of 200 μM for 24 h. Thereafter, cells were treated with 1 μM of Calcein AM and EthD-1. Magnification: X100. **Figure S7.** Cell viability assay of intestinal epithelial celltreated with GL, Lf, and Lf-GL, respectively.Optical image of Caco-2 cell that treated with GL equivalent concentration of 50 μM for 24 h. Scale bar: 500 μmCell viability of Caco-2 cell that treated with GL equivalent concentration of 25 μM to 100 μM for 24 h. Data were expressed as mean ± S.E.M. **Figure S8.** GBM spheroid growth inhibition of Lf-GL. To form U87MG spheroid, cells were seeded at density of 1X10^5^ cells in concave mold. After spheroids were formed, they were transferred to 24-well plate coated with 2% agarose gel. The constructed spheroids were treated with conditioned medium at 2 days interval after a washing step with PBS.Morphology of U87MG spheroid treated with Lf-GL of GL equivalent concentration of 50 μM on day 0, 2, 4 and 6, respectively. Scale bar: 200 μmGBM spheroids time-related volume after treated with Lf-GL of GL equivalent concentration of 50 μM. Data were expressed as mean ± S.E.M. **P <0.01 versus Control. **Figure S9.** Non-apoptotic GBM spheroid growth inhibition of Lf-GL. Live and dead staining of GBM spheroids on day 6 that treated with GL equivalent concentration of 50 μM. Scale bar: 200 μm. **Figure S10.** Non-apoptotic U87MG growth inhibition of Lf-GL. Annexin V-DY-634 / PI apoptosis staining to detect the apoptosis and necrosis in the Control, GL, Lf, and Lf-GL treated group at all GL equivalent concentration of 50 μM for 24 h. However, the cell population of annexin V/PIin the Lf-GL treatment group was 0.28%, which was even lower level to 1.40% in the Control group. **Figure S11.** Significant cellular uptake of Lf-GL in U87MG.Flow cytometry to evaluate cellular uptake of FITC tagged-Lf-GLin U87MG for 2,6, and 18 h. The 2 h pre-treatment of Lf and Pitstop® were treated in the concentration of 500 nM and 75 nM, respectively.Quantification of cellular uptake for 18 h. Data were expressed as mean ± S.E.M. **Figure S12.** Live and dead assay of HUVEC in the Control, GL, Lf, and Lf-GL group. The cells were treated with GL equivalent concentration of 100 μM. for 24 h. Thereafter, cells were treated with 1 μM of Calcein AM and EthD-1. Magnification: X100. **Figure S13.** HUVEC growth inhibition of Lf-GL. Annexin V-DY-634/PI apoptosis staining to detect the apoptosis and necrosis in the Control, GL, Lf, and Lf-GL treated group at all GL equivalent concentration of 100 μM for 24 h.** Figure S14.** Significant inhibitory effect of Lf-GL in aortic ring angiogenesis. Optical images of vessel regression in aortic ring with Lf-GL treatment. Aortic ring assay was carried out in 48-well plate coated with Matrigel. VEGF-non treated control represents that aortic ring incubated with normal medium. VEGF-treated control represents that aortic ring incubated with 25 ng mL^-1^ VEGF-conditioned medium. GL, Lf and Lf-GL groups represents that aortic ring incubated with VEGF-conditioned medium containing GL, Lf, and Lf-GL with GL equivalent concentration of 200 μM, respectively. Magnification: X100. Scale bar: 100 μm. **Figure S15.** Fluorescence tracer images of intravenously injected GL and Lf-GL. Balb/c mice were administered either FITC-tagged GL or FITC-tagged Lf-GL at a GL equivalent concentration of 50 mg kg^-1^ body weight via tail vein injection. The fluorescence signals of FITC-tagged GL or FITC-tagged Lf-GL in organs were imaged using an in vivo imaging system. The exposure time was fixed to 200 sec for analyzing fluorescent signals from tissues. The quantified fluorescence signal of brain at each time point was measured with Intensity Unit. **Figure S16.** Histopathological analysis of heart, liver, lung, spleen, and kidney after 28 doses. The tissues were histologically analyzed after intravenous administration of daily dosesto GBM-modeled mice for 28 days. Scale bar: 50 μm.

## Data Availability

The datasets used and/or analysed during the current study are available from the corresponding author on reasonable request.

## Abbreviations

HMGB1: High-mobility group box-1
DAMP: Damaged-associated molecular pattern
GL: Glycyrrhizin
PK: Pharmacokinetics
PD: Pharmacodynamics
Lf-GL: Lactoferrin-glycyrrhizin conjugate
SPR: Surface plasmon resonance
LfR: Lactoferrin receptor
GBM: Glioblastoma multiforme
VEGF: Vascular endothelial growth factor
RAGE: Receptor for advanced glycation end products
BBB: Blood-brain barrier
MALDI TOF: Matrix-assisted laser desorption/ionization time-of-flight
CCK-8: Cell counting kit-8
p-ERK: Phosphorylated-ERK
I.U.: Intensity units
RES: Reticuloendothelial system
MFI: Mean fluorescence intensity
CXCR-4: C-X-C motif chemokine receptor 4
K_D_: Equilibrium dissociation constant
PPI: Protein-protein interactions
TAMs: Tumor-associated macrophages
EMT: Epithelial mesenchymal transition

## References

[1] Hossain MJ, Xiao W, Tayeb M, Khan S. Epidemiology and prognostic factors of pediatric brain tumor survival in the US: Evidence from four decades of population data. Cancer Epidemiol. 2021;72:101942. 10.1016/j.canep.2021.101942 PMC8142618 33946020

[2] Dolecek TA, Propp JM, Stroup NE, Kruchko C. CBTRUS statistical report: Primary brain and central nervous system tumors diagnosed in the United States in 2005–2009. Neuro Oncol. 2012;14 SUPPL.5. 10.1093/neuonc/nos218 PMC3480240 23095881

[3] Batchelor TT, Reardon DA, De Groot JF, Wick W, Weller M (2014). Antiangiogenic therapy for glioblastoma: Current status and future prospects. Clin Cancer Res.

[4] Carmeliet P, Jain RK (2011). Principles and mechanisms of vessel normalization for cancer and other angiogenic diseases. Nat Rev Drug Discov.

[5] ER, HW, TT-W, GW, BF, JC, et al. The prognostic value of [123I]-vascular endothelial growth factor ([123I]-VEGF) in glioma. Eur J Nucl Med Mol Imaging. 2018;45:2396–403. 10.1007/s00259-018-4088-y PMC6208804 30062604

[6] Cohen MH, Shen YL, Keegan P, Pazdur R (2009). FDA Drug Approval Summary: Bevacizumab (Avastin®) as Treatment of Recurrent Glioblastoma Multiforme. Oncologist.

[7] Yu M, Wang J, Li W, Yuan YZ, Li CY, Qian XH (2008). Proteomic screen defines the hepatocyte nuclear factor 1α-binding partners and identifies HMGB1 as a new cofactor of HNF1α. Nucleic Acids Res.

[8] Ulloa L, Messmer D (2006). High-mobility group box 1 (HMGB1) protein: Friend and foe. Cytokine Growth Factor Rev.

[9] Angelopoulou E, Piperi C, Adamopoulos C, Papavassiliou AG (2016). Pivotal role of high-mobility group box 1 (HMGB1) signaling pathways in glioma development and progression. J Mol Med.

[10] Cheng P, Ma Y, Gao Z, Duan L (2018). High mobility group box 1 (HMGB1) predicts invasion and poor prognosis of glioblastoma multiforme via activating AKT signaling in an autocrine pathway. Med Sci Monit.

[11] Wang XJ, Zhou SL, Fu XD, Liang B, Shou JX, Wang JY (2015). Clinical and prognostic significance of high-mobility group box-1 in human gliomas. Exp Ther Med.

[12] He H, Wang X, Chen J, Sun L, Sun H, Xie K (2019). High-mobility group box 1 (HMGB1) promotes angiogenesis and tumor migration by regulating hypoxia-inducible factor 1 (HIF-1α) expression via the phosphatidylinositol 3-kinase (PI3K)/AKT signaling pathway in breast cancer cells. Med Sci Monit.

[13] Mollica L, De Marchis F, Spitaleri A, Dallacosta C, Pennacchini D, Zamai M (2007). Glycyrrhizin Binds to High-Mobility Group Box 1 Protein and Inhibits Its Cytokine Activities. Chem Biol.

[14] Kim KJ, Choi JS, Kim KW, Jeong JW (2013). The anti-angiogenic activities of glycyrrhizic acid in tumor progression. Phyther Res.

[15] Bonafé GA, dos Santos JS, Ziegler JV, Umezawa K, Ribeiro ML, Rocha T, et al. Growth inhibitory effects of dipotassium glycyrrhizinate in glioblastoma cell lines by targeting microRNAs through the NF-κB signaling pathway. Front Cell Neurosci. 2019;13:1–14. 10.3389/fncel.2019.00216 PMC6546822 31191251

[16] Kim HS, Lee SJ, Lee DY (2021). Milk protein-shelled gold nanoparticles with gastrointestinally active absorption for aurotherapy to brain tumor. Bioact Mater.

[17] Kim HS, Seo M, Park TE, Lee DY (2022). A novel therapeutic strategy of multimodal nanoconjugates for state-of-the-art brain tumor phototherapy. J Nanobiotechnol.

[18] Hegde MM, Prabhu S, Mutalik S, Chatterjee A, Goda JS, Satish Rao BS (2022). Multifunctional lipidic nanocarriers for effective therapy of glioblastoma: recent advances in stimuli-responsive, receptor and subcellular targeted approaches. J Pharm Investig.

[19] Ji B, Maeda J, Higuchi M, Inoue K, Akita H, Harashima H (2006). Pharmacokinetics and brain uptake of lactoferrin in rats. Life Sci.

[20] Su Z, Xing L, Chen Y, Xu Y, Yang F, Zhang C (2014). Lactoferrin-modified poly(ethylene glycol)-grafted BSA nanoparticles as a dual-targeting carrier for treating brain gliomas. Mol Pharm.

[21] Huang RQ, Ke WL, Qu YH, Zhu JH, Pei YY, Jiang C (2007). Characterization of lactoferrin receptor in brain endothelial capillary cells and mouse brain. J Biomed Sci.

[22] Emmerich CH, Gamboa LM, Hofmann MCJ, Bonin-Andresen M, Arbach O, Schendel P (2021). Improving target assessment in biomedical research: the GOT-IT recommendations. Nat Rev Drug Discov.

[23] Shi L, Tang C, Yin C (2012). Glycyrrhizin-modified O-carboxymethyl chitosan nanoparticles as drug vehicles targeting hepatocellular carcinoma. Biomaterials.

[24] Shankaranarayanan JS, Kanwar JR, Al-Juhaishi AJA, Kanwar RK. Doxorubicin conjugated to immunomodulatory anticancer lactoferrin displays improved cytotoxicity overcoming prostate cancer chemo resistance and inhibits tumour development in TRAMP mice. Sci Rep. 2016;6:32062. 10.1038/srep32062 PMC5005995 27576789

[25] Li Y jun, Wang L, Zhang B, Gao F, Yang CM. Glycyrrhizin, an HMGB1 inhibitor, exhibits neuroprotective effects in rats after lithium-pilocarpine-induced status epilepticus. J Pharm Pharmacol. 2019;71:390–9. 10.1111/jphp.13040 30417405

[26] Smolarczyk R, Cichoń T, Matuszczak S, Mitrus I, Lesiak M, Kobusińska M (2012). The role of glycyrrhizin, an inhibitor of HMGB1 protein, in anticancer therapy. Arch Immunol Ther Exp (Warsz).

[27] Mollica L, Morra G, Colombo G, Musco G (2011). HMGB1-carbenoxolone interactions: Dynamics insights from combined nuclear magnetic resonance and molecular dynamics. Chem Asian J.

[28] Fang H, Zhao X, Lin Y, Yang S, Hu J (2018). A Natural Glycyrrhizic Acid-Tailored Light-Responsive Gelator. Chem Asian J.

[29] Oraiopoulou ME, Tzamali E, Tzedakis G, Vakis A, Papamatheakis J, Sakkalis V. In vitro/in silico study on the role of doubling time heterogeneity among primary glioblastoma cell lines. Biomed Res Int. 2017;2017:8569328. 10.1155/2017/8569328 PMC5684616 29226151

[30] Wong C, Stylianopoulos T, Cui J, Martin J, Chauhan VP, Jiang W (2011). Multistage nanoparticle delivery system for deep penetration into tumor tissue. Proc Natl Acad Sci U S A.

[31] Hwang HH, Kim HS, Lee DY. Gastrointestinally absorbable lactoferrin-heparin conjugate with anti-angiogenic activity for treatment of brain tumor. J Control Release. 2023;355:730–44. 10.1016/j.jconrel.2023.02.002 36764526

[32] Kuo YC, Chen YC (2015). Targeting delivery of etoposide to inhibit the growth of human glioblastoma multiforme using lactoferrin- and folic acid-grafted poly(lactide-co-glycolide) nanoparticles. Int J Pharm.

[33] Bassi R, Giussani P, Anelli V, Colleoni T, Pedrazzi M, Patrone M (2008). HMGB1 as an autocrine stimulus in human T98G glioblastoma cells: Role in cell growth and migration. J Neurooncol.

[34] Iularo M, Scatena M, Zhu WH, Fogel E, Wieting SL, Nicosia RF (2003). Rat aorta-derived mural precursor cell express the Tie2 receptor and respond directly to stimulation by angiopoietins. J Cell Sci.

[35] Schneider SW, Ludwig T, Tatenhorst L, Braune S, Oberleithner H, Senner V (2004). Glioblastoma cells release factors that disrupt blood-brain barrier features. Acta Neuropathol.

[36] Cho HJ (2020). Recent progresses in the development of hyaluronic acid-based nanosystems for tumor-targeted drug delivery and cancer imaging. J Pharm Investig.

[37] Kramer JA, O’neill E, Phillips ME, Bruce D, Smith T, Albright MM, et al. Early Toxicology Signal Generation in the Mouse. Toxicol Pathol. 2010;38:452–71. 10.1177/0192623310364025 20305093

[38] Bakr AF, Shao P, Farag MA (2021). Recent advances in glycyrrhizin metabolism, health benefits, clinical effects and drug delivery systems for efficacy improvement; a comprehensive review. Phytomedicine.

[39] Chavakis E, Hain A, Vinci M, Carmona G, Bianchi ME, Vajkoczy P (2007). High-mobility group box 1 activates integrin-dependent homing of endothelial progenitor cells. Circ Res.

[40] Igney FH, Krammer PH (2002). Death and anti-death: Tumour resistance to apoptosis. Nat Rev Cancer.

[41] Lawrence MD, Blyth BJ, Ormsby RJ, Tilley WD, Sykes PJ (2013). False-positive TUNEL staining observed in SV40 based transgenic murine prostate cancer models. Transgenic Res.

[42] Mirzayans R, Murray D (2020). Do TUNEL and other apoptosis assays detect cell death in preclinical studies?. Int J Mol Sci.

[43] Huang CY, Chiang SF, Chen WTL, Ke TW, Chen TW, You YS (2018). HMGB1 promotes ERK-mediated mitochondrial Drp1 phosphorylation for chemoresistance through RAGE in colorectal cancer. Cell Death Dis.

[44] Okui T, Hiasa M, Ryumon S, Ono K, Kunisada Y, Ibaragi S (2021). The HMGB1/RAGE axis induces bone pain associated with colonization of 4T1 mouse breast cancer in bone. J Bone Oncol.

[45] Xu W, Qian J, Zeng F, Li S, Guo W, Chen L (2019). Protein kinase Ds promote tumor angiogenesis through mast cell recruitment and expression of angiogenic factors in prostate cancer microenvironment. J Exp Clin Cancer Res.

[46] Feng T, Yu H, Xia Q, Ma Y, Yin H, Shen Y (2017). Cross-talk mechanism between endothelial cells and hepatocellular carcinoma cells via growth factors and integrin pathway promotes tumor angiogenesis and cell migration. Oncotarget.

[47] Pereira CS, Guedes JP, Gonçalves M, Loureiro L, Castro L, Gerós H (2016). Lactoferrin selectively triggers apoptosis in highly metastatic breast cancer cells through inhibition of plasmalemmal V-H+-ATPase. Oncotarget.

[48] Farooqui A, Khan F, Khan I, Ansari IA (2017). Glycyrrhizin induces reactive oxygen species-dependent apoptosis and cell cycle arrest at G0/G1 in HPV18+ human cervical cancer HeLa cell line. Biomed Pharmacother.

[49] Moser B, Janik S, Schiefer AI, Müllauer L, Bekos C, Scharrer A, et al. Expression of RAGE and HMGB1 in thymic epithelial tumors, thymic hyperplasia and regular thymic morphology. PLoS One. 2014;9:e94118. 10.1371/journal.pone.0094118 PMC3976415 24705787

[50] Elass-Rochard E, Legrand D, Salmon V, Roseanu A, Trif M, Tobias PS (1998). Lactoferrin inhibits the endotoxin interaction with CD14 by competition with the lipopolysaccharide-binding protein. Infect Immun.

[51] Kell DB, Heyden EL, Pretorius E. The Biology of Lactoferrin, an Iron-Binding Protein That Can Help Defend Against Viruses and Bacteria. Front Immunol. 2020;11:1–15. 10.3389/fimmu.2020.01221 PMC7271924 32574271

[52] Akiyama Y, Oshima K, Kuhara T, Shin K, Abe F, Iwatsuki K (2013). A lactoferrin-receptor, intelectin 1, affects uptake, sub-cellular localization and release of immunochemically detectable lactoferrin by intestinal epithelial Caco-2 cells. J Biochem.

[53] Ando K, Hasegawa K, Shindo KI, Furusawa T, Fujino T, Kikugawa K (2010). Human lactoferrin activates NF-κB through the Toll-like receptor 4 pathway while it interferes with the lipopolysaccharide-stimulated TLR4 signaling. FEBS J.

[54] Takayama Y, Aoki R, Uchida R, Tajima A, Aoki-Yoshida A (2017). Role of CXC chemokine receptor type 4 as a lactoferrin receptor. Biochem Cell Biol.

[55] Elzoghby AO, Abdelmoneem MA, Hassanin IA, Abd Elwakil MM, Elnaggar MA, Mokhtar S, et al. Lactoferrin, a multi-functional glycoprotein: Active therapeutic, drug nanocarrier & targeting ligand. Biomaterials. 2020;263:120355. 10.1016/j.biomaterials.2020.120355 PMC7480805 32932142

[56] Kaksonen M, Roux A (2018). Mechanisms of clathrin-mediated endocytosis. Nat Rev Mol Cell Biol.

[57] Kang R, Xie Y, Zhang Q, Hou W, Jiang Q, Zhu S (2017). Intracellular HMGB1 as a novel tumor suppressor of pancreatic cancer. Cell Res.

[58] He S, Cheng J, Sun L, Wang Y, Wang C, Liu X (2018). HMGB1 released by irradiated tumor cells promotes living tumor cell proliferation via paracrine effect article. Cell Death Dis.

[59] Kang HJ, Lee H, Choi HJ, Youn JH, Shin JS, Ahn YH (2009). Non-histone nuclear factor HMGB1 is phosphorylated and secreted in colon cancers. Lab Investig.

[60] Smolarczyk R, Cichoń T, Jarosz M, Szala S (2012). HMGB1 – rola w progresji i terapii przeciwnowotworowej. Postep Hig Med Dosw.

[61] Van Beijnum JR, Nowak-Sliwinska P, Van Den Boezem E, Hautvast P, Buurman WA, Griffioen AW (2013). Tumor angiogenesis is enforced by autocrine regulation of high-mobility group box 1. Oncogene.

[62] Chung HW, Lim JB (2017). High-mobility group box-1 contributes tumor angiogenesis under interleukin-8 mediation during gastric cancer progression. Cancer Sci.

[63] Bumbaca B, Li Z, Shah DK (2019). Pharmacokinetics of protein and peptide conjugates. Drug Metab Pharmacokinet.

[64] Kurzrock R, Gabrail N, Chandhasin C, Moulder S, Smith C, Brenner A (2012). Safety, pharmacokinetics, and activity of GRN1005, a novel conjugate of angiopep-2, a peptide facilitating brain penetration, and paclitaxel, in patients with advanced solid tumors. Mol Cancer Ther.

[65] Younes A, Aggarwall BB (2003). Clinical implications of the tumor necrosis factor family in benign and malignant hematologic disorders: A short review. Cancer.

[66] Lee SY, Ju MK, Jeon HM, Jeong EK, Lee YJ, Kim CH, et al. Regulation of tumor progression by programmed necrosis. Oxid Med Cell Longev. 2018;2018:3537471. 10.1155/2018/3537471 PMC5831895 29636841

[67] Jube S, Rivera ZS, Bianchi ME, Powers A, Wang E, Pagano I (2012). Cancer cell secretion of the DAMP protein HMGB1 supports progression in malignant mesothelioma. Cancer Res.

[68] Zhu J, Huang R, Yang R, Xiao Y, Yan J, Zheng C (2021). Licorice extract inhibits growth of non-small cell lung cancer by down-regulating CDK4-Cyclin D1 complex and increasing CD8+ T cell infiltration. Cancer Cell Int.

[69] Siqueiros-Cendón T, Arévalo-Gallegos S, Iglesias-Figueroa BF, García-Montoya IA, Salazar-Martínez J, Rascón-Cruz Q (2014). Immunomodulatory effects of lactoferrin. Acta Pharmacol Sin.

[70] Lee WS, Yang H, Chon HJ, Kim C (2020). Combination of anti-angiogenic therapy and immune checkpoint blockade normalizes vascular-immune crosstalk to potentiate cancer immunity. Exp Mol Med.

[71] Song Y, Fu Y, Xie Q, Zhu B, Wang J, Zhang B. Anti-angiogenic Agents in Combination With Immune Checkpoint Inhibitors: A Promising Strategy for Cancer Treatment. Front Immunol. 2020;11:1–17. 10.3389/fimmu.2020.01956 PMC7477085 32983126

