# Supplementary material for: Inhibition of DAMP actions in the tumoral microenvironment using lactoferrin-glycyrrhizin conjugate for glioblastoma therapy
Source: Biomater Res. 2023 May 20;27:52. doi: 10.1186/s40824-023-00391-w (PMC10200060; doi:10.1186/s40824-023-00391-w)
Supplement: Supplementary file 1 — Additional file 1: Figure S1. FT-IR spectra of GL, Lf and Lf-GL respectively. The conjugation between GL and Lf was confirmed by Fourier transform infraredanalysis. The amide I and amide II vibrations observed at 1,523 cm-1 and 1,630 cm-1 in both Lf and Lf-GL. The 1,035 cm-1 indicates primary alcohol stretch of GL. Figure S2. SDS-PAGE result of Lf and Lf-GL. The successful synthesis of Lf-GL could be assumed from the sodium dodecyl sulfate polyacrylamide gel electrophoresisresults as a molecular weightrise in the band above 80 kDa, the MW of native Lf. Figure S3. HPLC result of GL and Lf-GL. High performance liquid chromatographyusing gel permeation chromatographycolumn was performed to verify GL content in Lf-GL conjugate. Mobile phase is composed of methanol, acetonitrile, water, and acetic acid in a ratio of 55:23.69:19.63:0.68. GL was dissolved in mobile phase in different concentrationand Lf-GLwas also dissolved in mobile phase. Ultrahydrogel 120 Columnwas used as column, flow rate was 1 mL min-1. Then, absorbance was measured in 254 nm and results were calibrated with Empower software. Figure S4. MALDI-TOF result of Lf and Lf-GL. MALDI-TOF was conducted at Seoul National Universityusing MALDI-TOF Voyager DE-STRand sinapinic acidaqueous solution containing about 30% acetonitrile in 0.15% trifluoroacetic acidwas used as a matrix. Average molecular weight of Lf-GL is 86,727.3 ± 98.1 Da. Considering the molecular weight of Lf is 78,497.9 Da and GL is 822.9 Da, binding ratio of Lf:GL is 1:9.6. Figure S5. Binding affinity against HMGB1. Surface Plasmon Resonancewas conducted at WoojungBSC. The Reichert SR7500DC systemand Scrubber2 softwarewere used and the CMDH chipwas used for immobilization of the human recombinant HMGB1 protein. HMGB1 protein was used as ligand and analytes were HMGB1 antibody, GL, Lf and Lf-GL conjugate. Immobilization buffer was 10 mM S.A.and running buffer was 1× PBS. The flow rate of the analyte was 30 μL min-1. The association and dissociati [file 40824_2023_391_MOESM1_ESM.docx]

Supplementary Materials for

**Inhibition of DAMP Actions in the Tumoral Microenvironment Using Lactoferrin-Glycyrrhizin Conjugate for Glioblastoma Therapy**

Hyung Shik Kim *et al.* and Dong Yun Lee*

*Corresponding author. Email: dongyunlee@hanyang.ac.kr

**This PDF file includes:**

Supplementary Text

Figs. S1 to S16


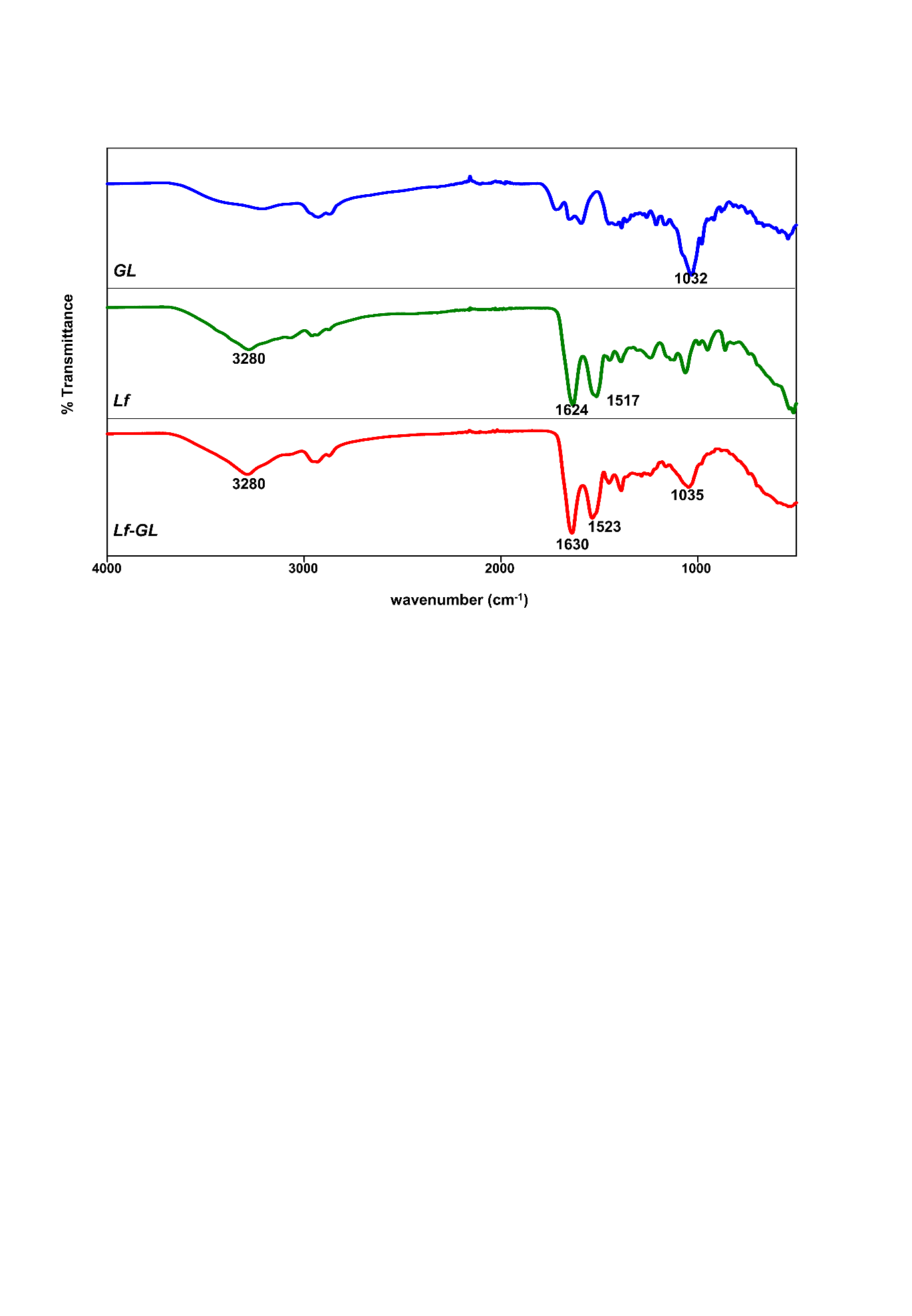


**Fig. S1. FT-IR spectra of GL, Lf and Lf-GL respectively.** The conjugation between GL and Lf was confirmed by Fourier transform infrared (FT-IR, NICOLET IS50, Thermo Fisher Scientific) analysis. The amide I and amide II vibrations observed at 1,523 cm^-1^ and 1,630 cm^-1^ in both Lf and Lf-GL. The 1,035 cm^-1^ indicates primary alcohol stretch of GL.


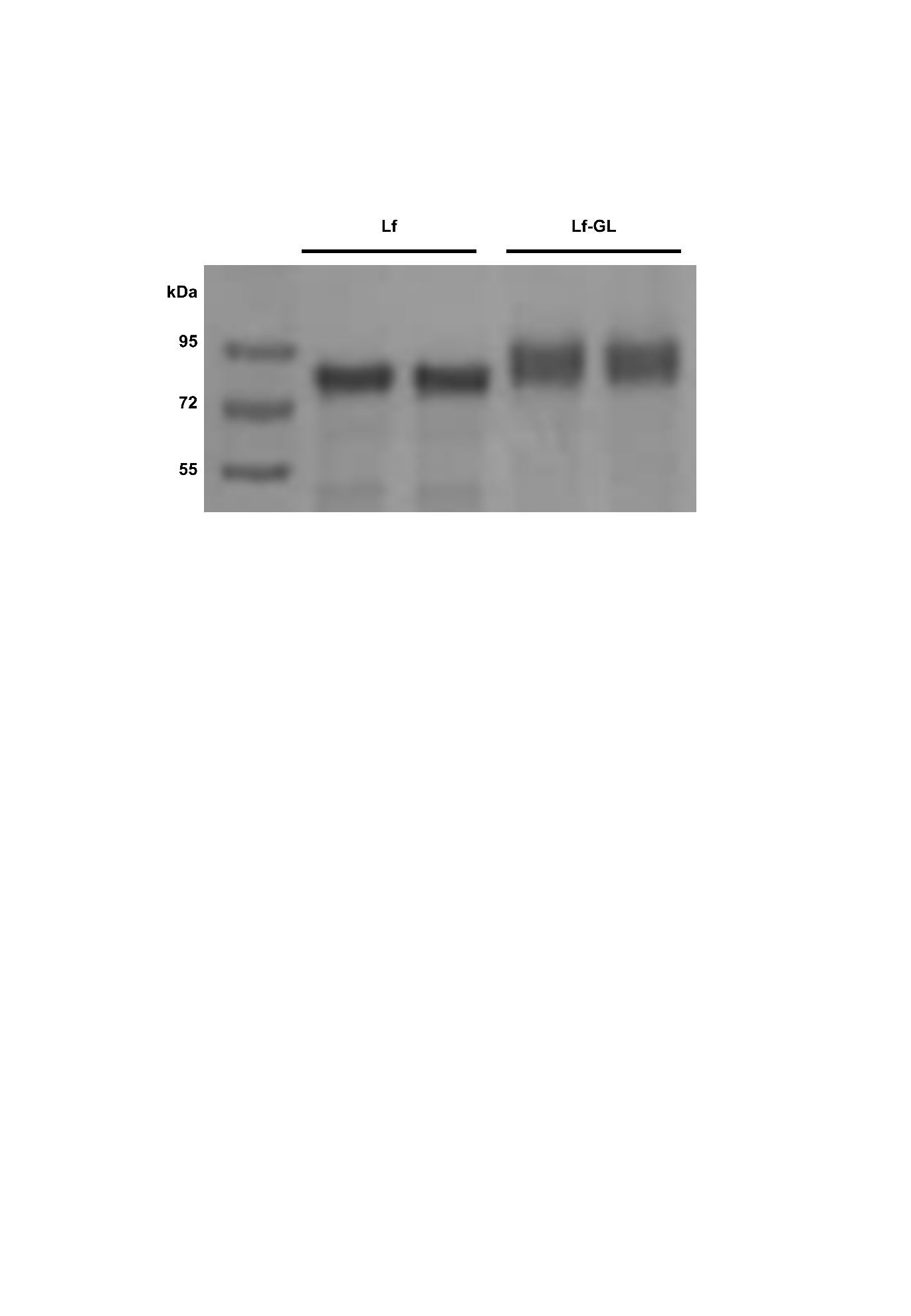


**Fig. S2. SDS-PAGE result of Lf and Lf-GL.** The successful synthesis of Lf-GL could be assumed from the sodium dodecyl sulfate polyacrylamide gel electrophoresis (SDS-PAGE) results as a molecular weight (MW) rise in the band above 80 kDa, the MW of native Lf.


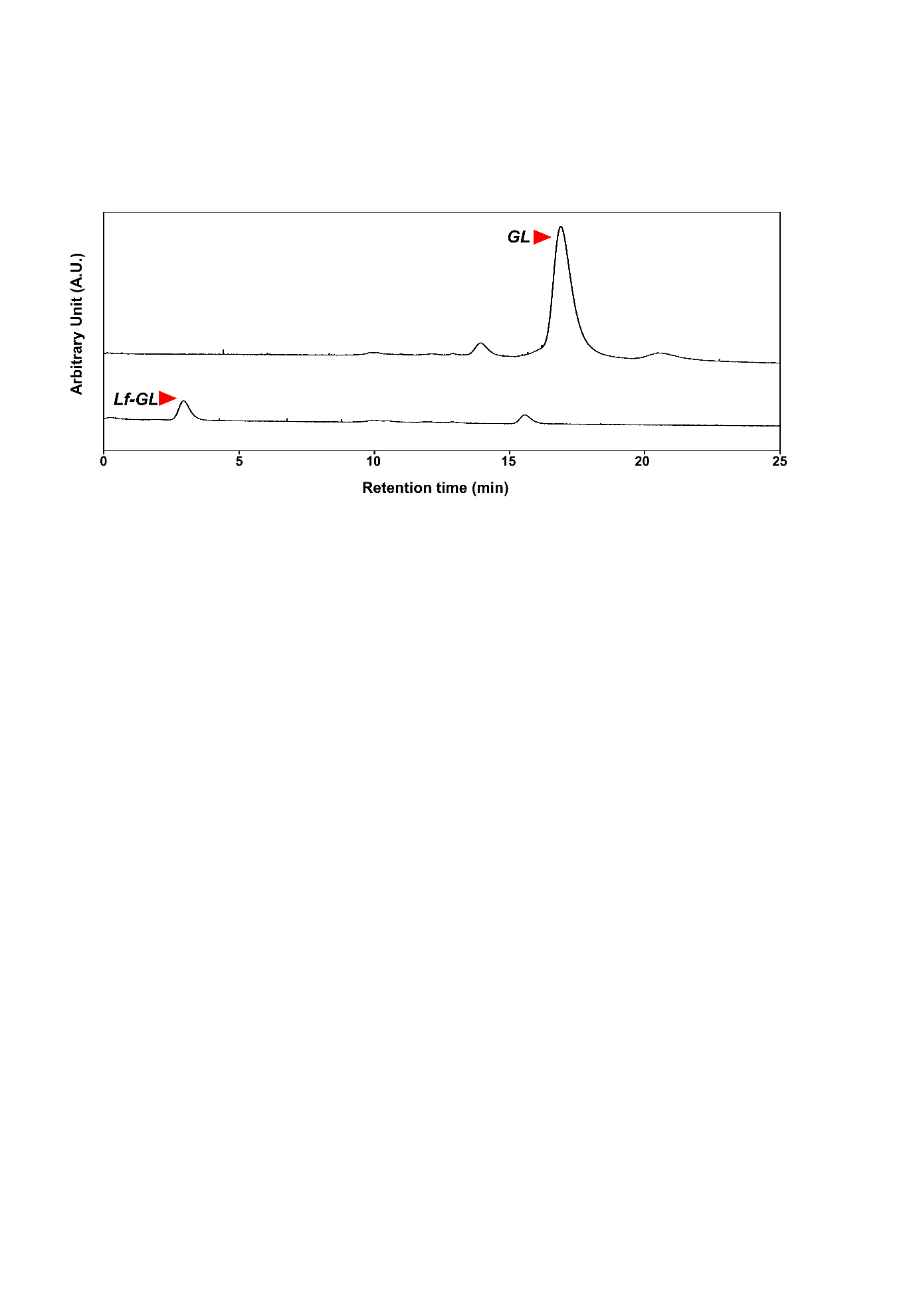


**Fig. S3. HPLC result of GL and Lf-GL.** High performance liquid chromatography (Alliance HPLC e2695, Waters, MA, UK) using gel permeation chromatography (GPC) column was performed to verify GL content in Lf-GL conjugate. Mobile phase is composed of methanol, acetonitrile, water, and acetic acid in a ratio of 55:23.69:19.63:0.68. GL was dissolved in mobile phase in different concentration (2.5 to 200 μM) and Lf-GL (25 μM) was also dissolved in mobile phase. Ultrahydrogel 120 Column (Waters) was used as column, flow rate was 1 mL min^-1^. Then, absorbance was measured in 254 nm and results were calibrated with Empower software (Waters).


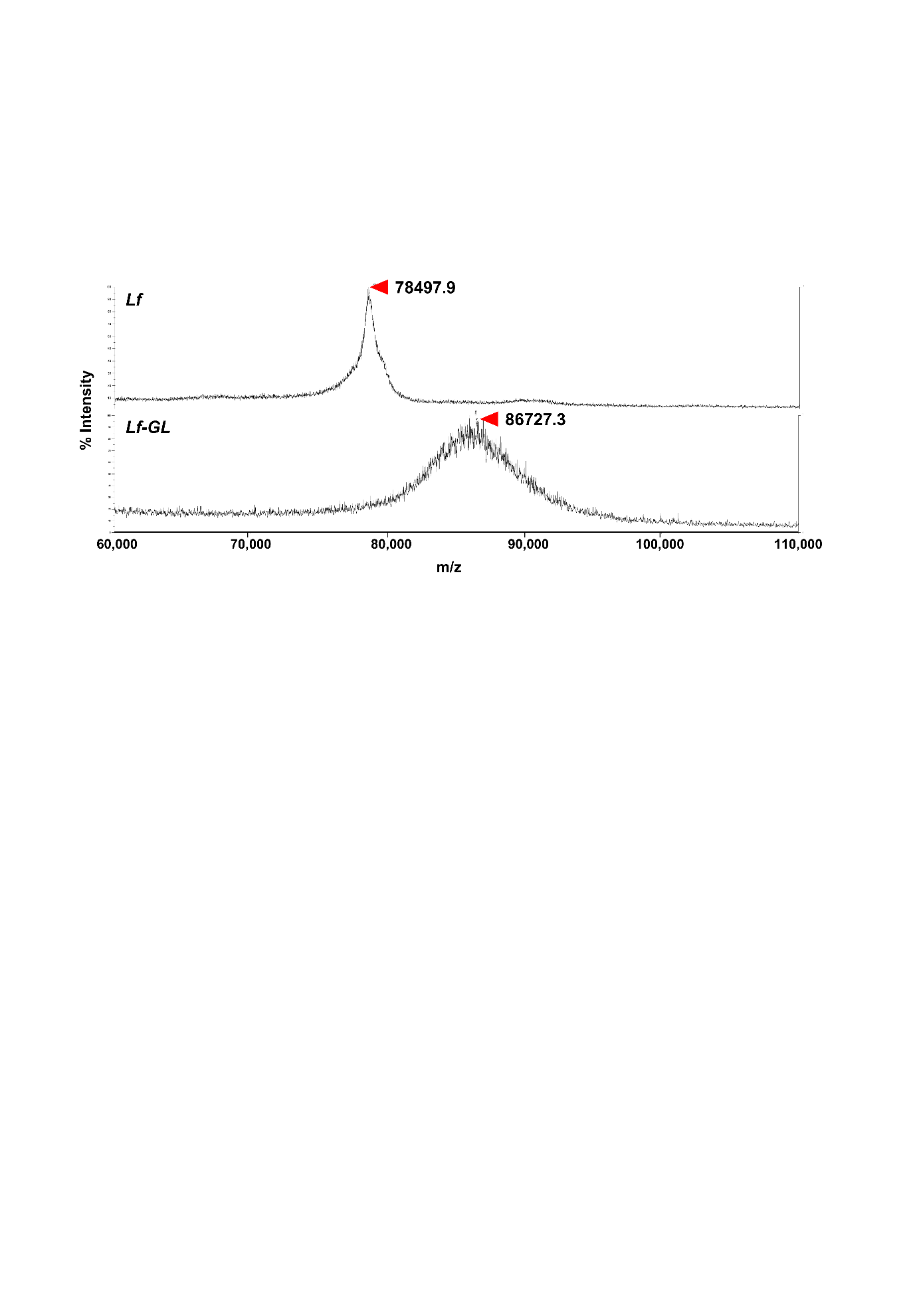


**Fig. S4. MALDI-TOF result of Lf and Lf-GL.** MALDI-TOF was conducted at Seoul National University (Korea) using MALDI-TOF Voyager DE-STR (Applied Biosystems, MA, USA) and sinapinic acid (Sigma-Aldrich) aqueous solution containing about 30% acetonitrile in 0.15% trifluoroacetic acid (Millipore) was used as a matrix. Average molecular weight of Lf-GL is 86,727.3 ± 98.1 Da. Considering the molecular weight of Lf is 78,497.9 Da and GL is 822.9 Da, binding ratio of Lf:GL is 1:9.6.


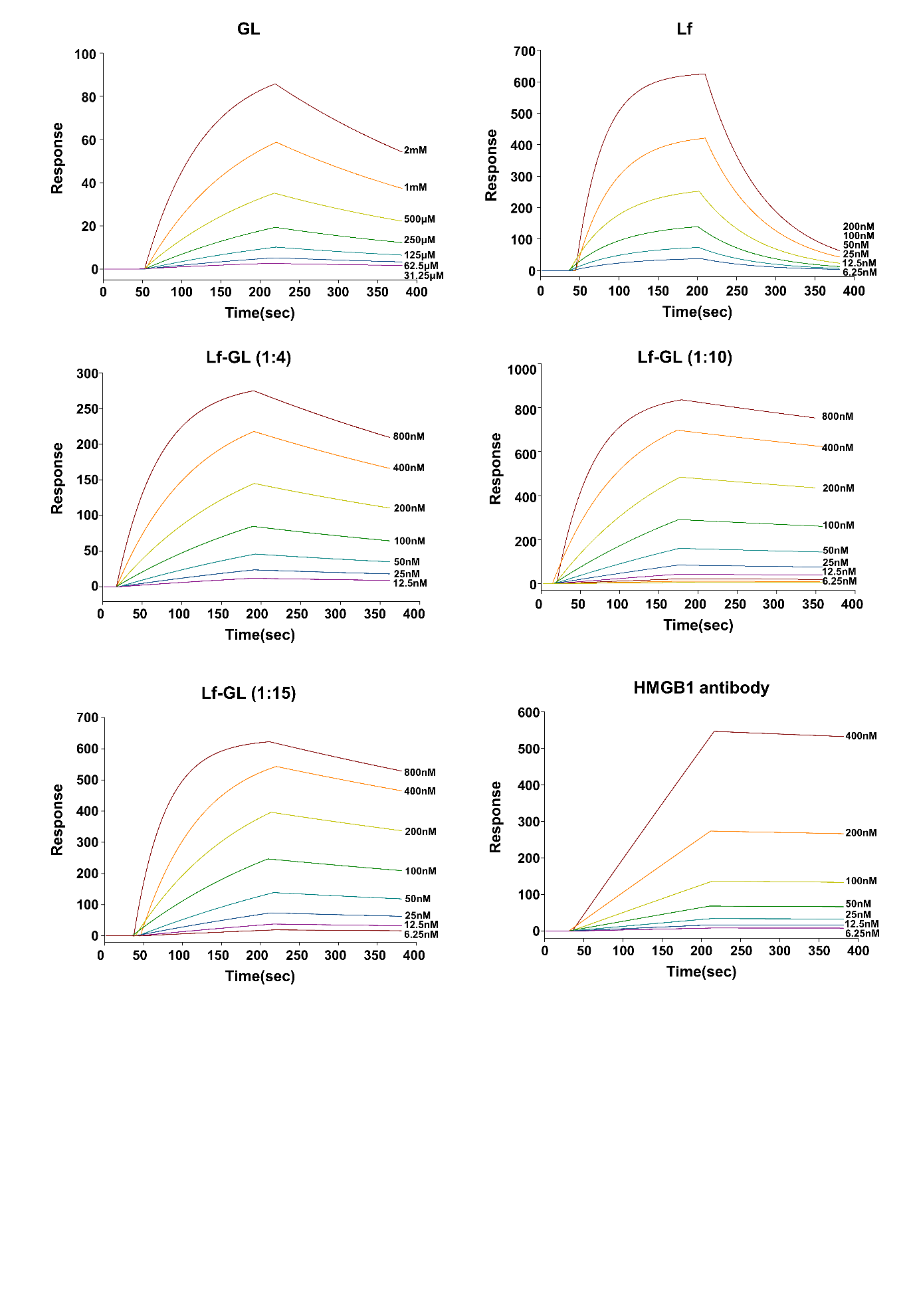


**Fig. S5. Binding affinity against HMGB1.** Surface Plasmon Resonance (SPR) was conducted at WoojungBSC (Korea). The Reichert SR7500DC system (Reichert technologies, NA, USA) and Scrubber2 software(Biologic software, FRA) were used and the CMDH chip (Cat #: 13206066, Reichert technologies) was used for immobilization of the human recombinant HMGB1 protein (Abcam). HMGB1 protein was used as ligand and analytes were HMGB1 antibody (Abcam), GL, Lf and Lf-GL conjugate. Immobilization buffer was 10 mM S.A. (pH 4.5) and running buffer was 1× PBS. The flow rate of the analyte was 30 μL min^-1^. The association and dissociation time were 3 min each and conducted at room temperature. Results are expressed in response units (RU) over time.


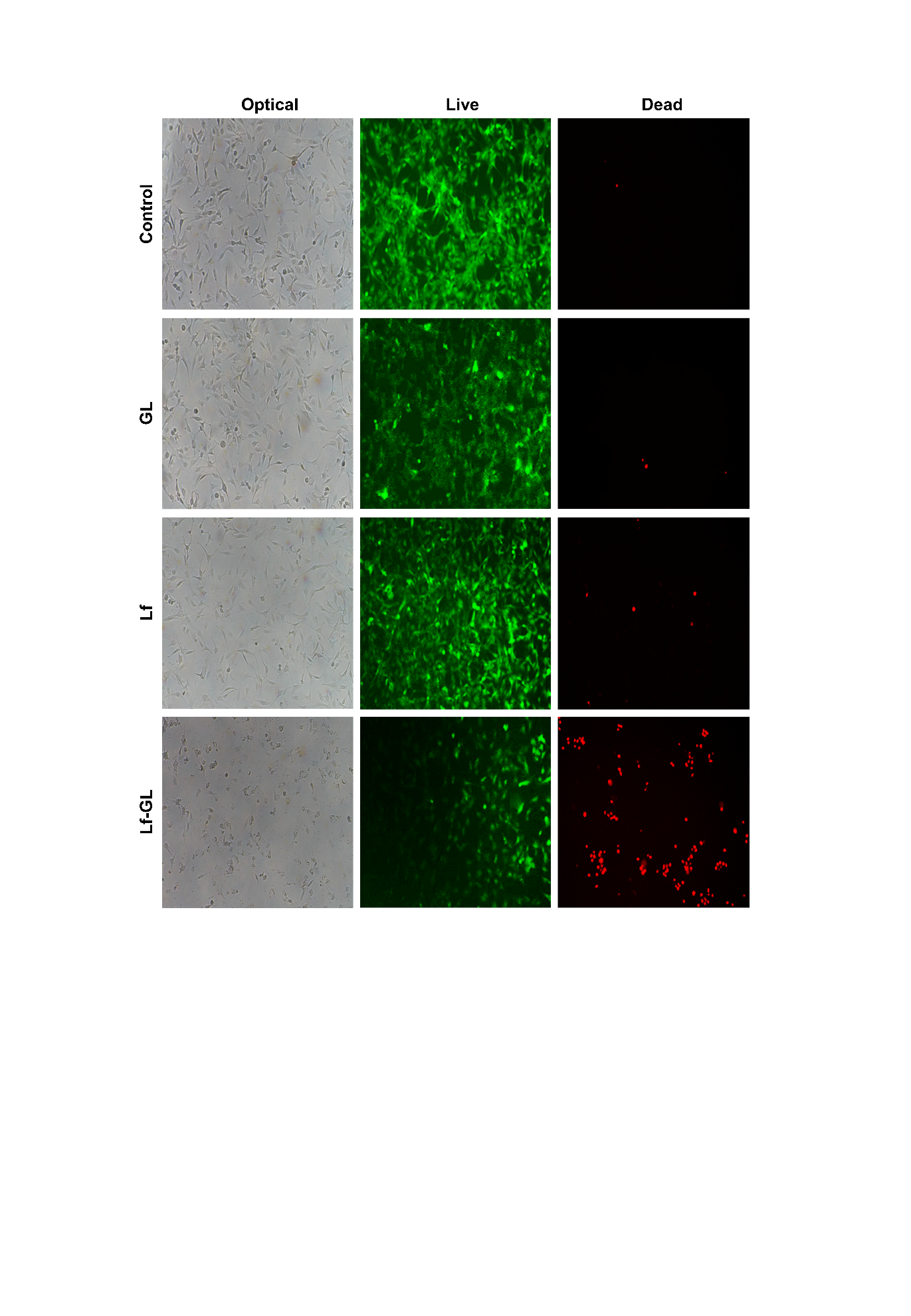


**Fig. S6. Live and dead assay of U87MG in the Control, GL, Lf, and Lf-GL group.** The cells were treated with GL equivalent concentration of 200 μM for 24 h. Thereafter, cells were treated with 1 μM of Calcein AM and EthD-1. Magnification: X100.


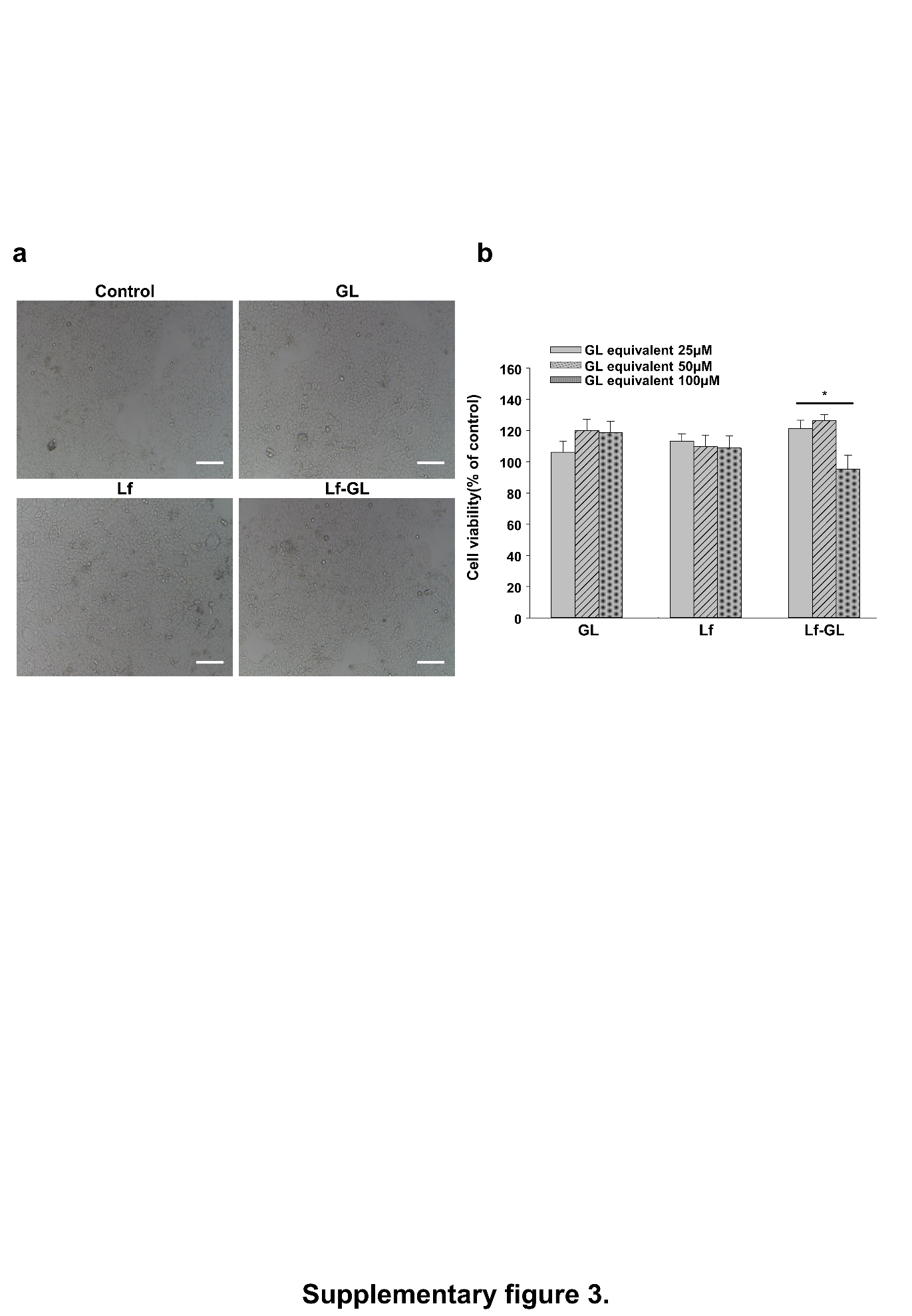


**Fig. S7. Cell viability assay of intestinal epithelial cell (Caco-2) treated with GL, Lf, and Lf-GL, respectively.** (a) Optical image of Caco-2 cell that treated with GL equivalent concentration of 50 μM for 24 h. Scale bar: 500 μm (b) Cell viability of Caco-2 cell that treated with GL equivalent concentration of 25 μM to 100 μM for 24 h. Data were expressed as mean ± S.E.M (n=5).


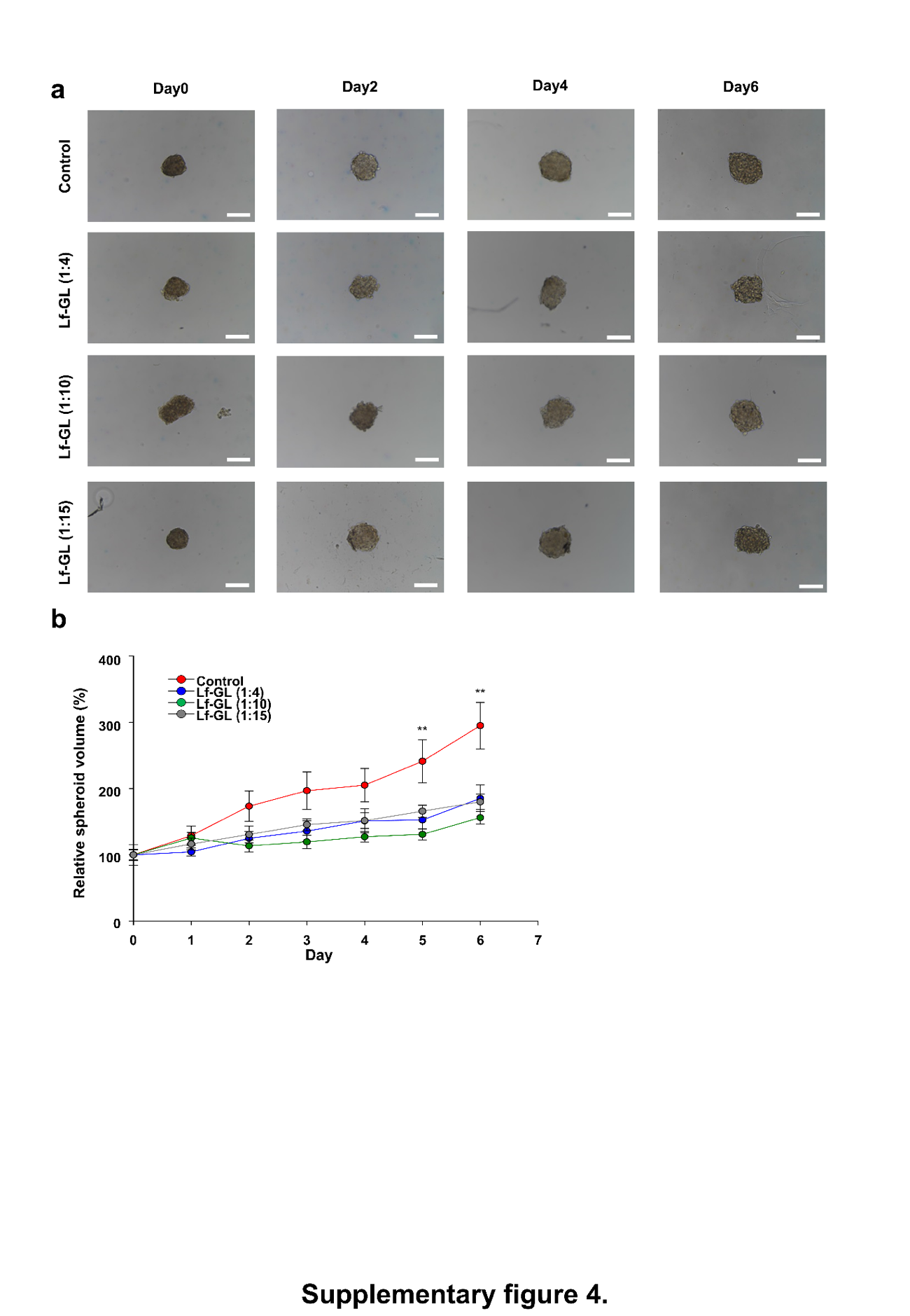


**Fig. S8. GBM spheroid growth inhibition of Lf-GL.** To form U87MG spheroid, cells were seeded at density of 1X10^5^ cells in concave mold (StemFIT 3D; MICROFIT, Korea). After spheroids were formed, they were transferred to 24-well plate coated with 2% agarose gel. The constructed spheroids were treated with conditioned medium at 2 days interval after a washing step with PBS. (a) Morphology of U87MG spheroid treated with Lf-GL of GL equivalent concentration of 50 μM on day 0, 2, 4 and 6, respectively. Scale bar: 200 μm (b) GBM spheroids time-related volume after treated with Lf-GL of GL equivalent concentration of 50 μM. Data were expressed as mean ± S.E.M (n=9). **P <0.01 versus Control.


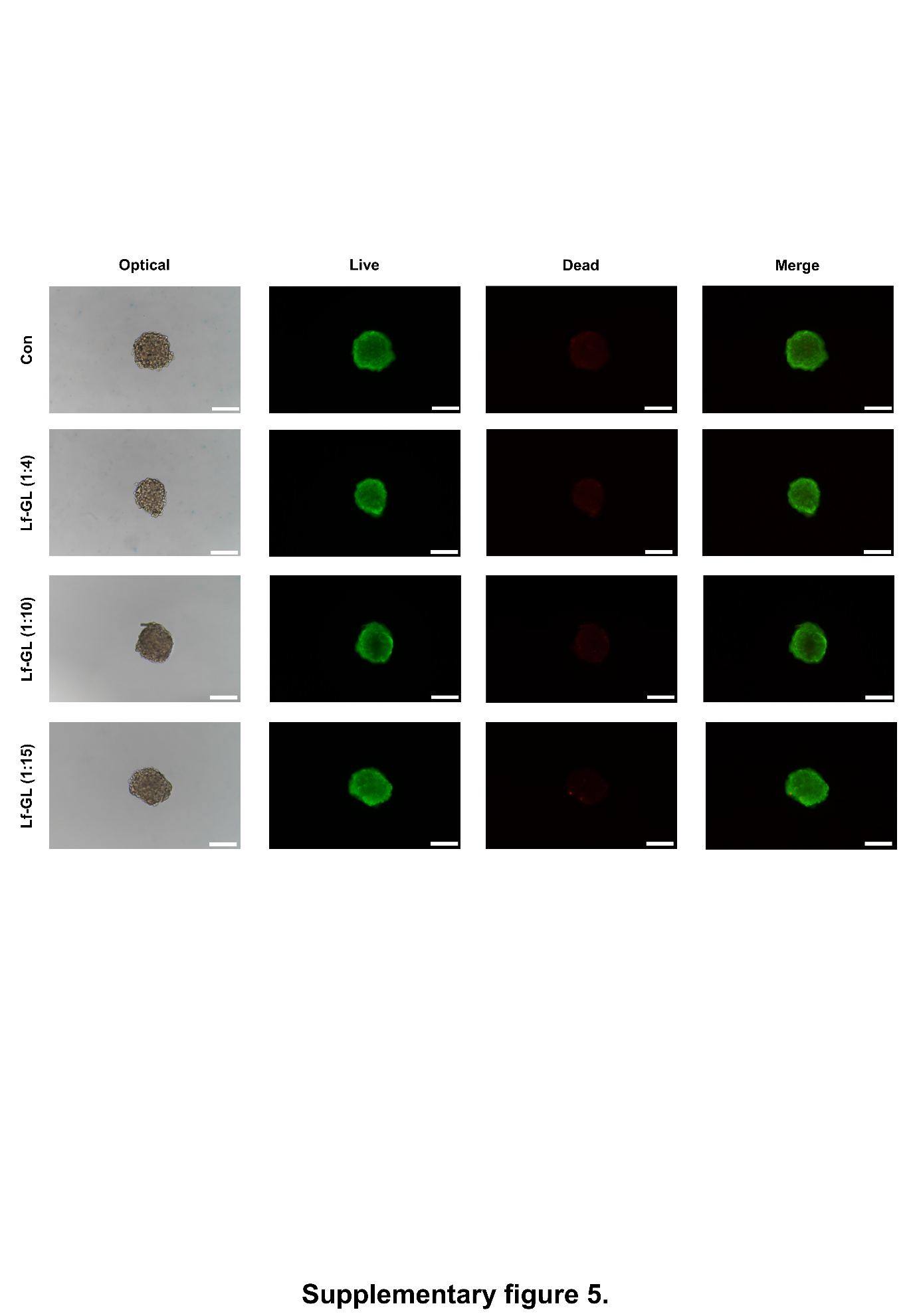


**Fig. S9. Non-apoptotic GBM spheroid growth inhibition of Lf-GL.** Live and dead staining of GBM spheroids on day 6 that treated with GL equivalent concentration of 50 μM. Scale bar: 200 μm.


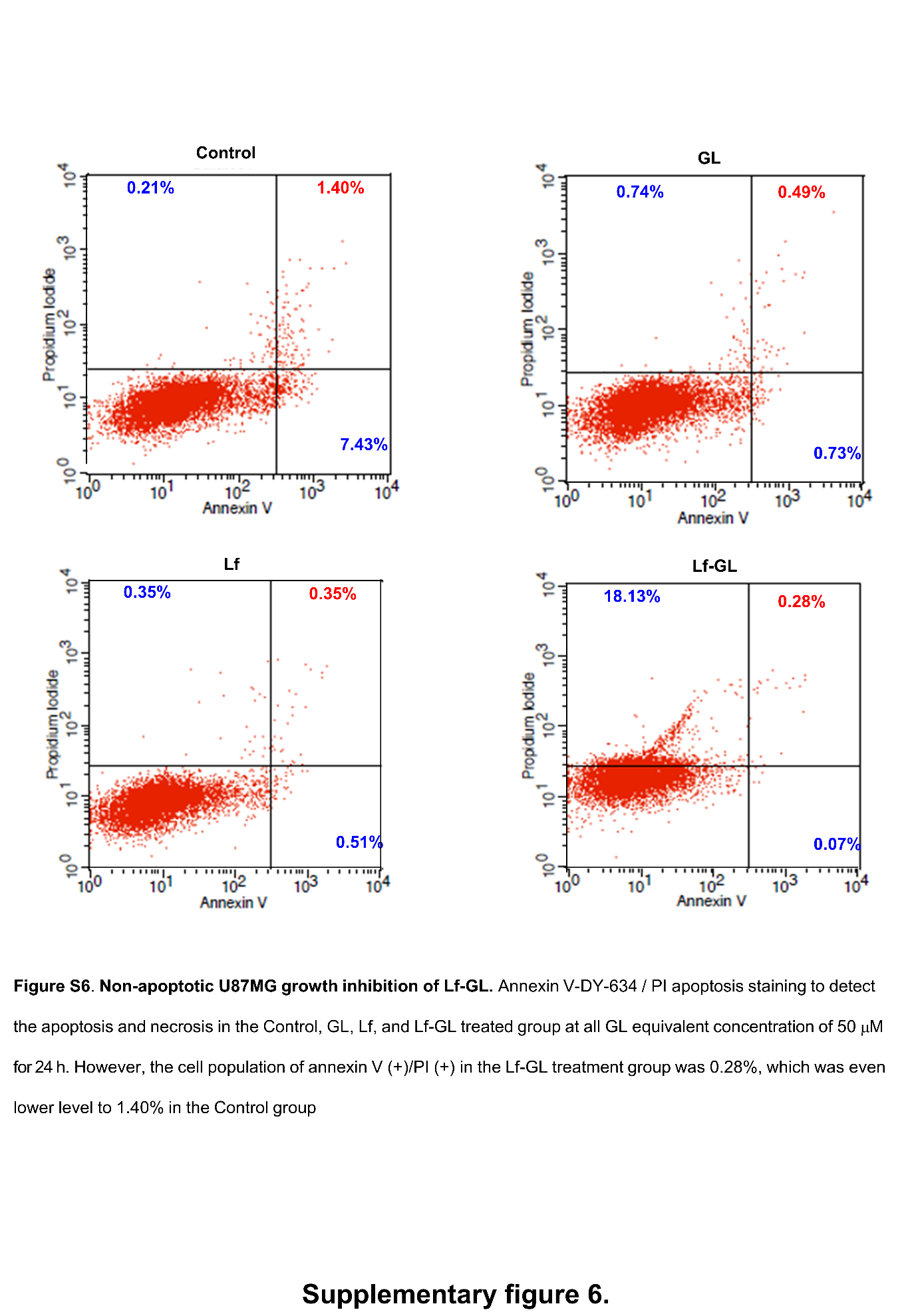


**Fig. S10. Non-apoptotic U87MG growth inhibition of Lf-GL.** Annexin V-DY-634 / PI apoptosis staining to detect the apoptosis and necrosis in the Control, GL, Lf, and Lf-GL treated group at all GL equivalent concentration of 50 μM for 24 h. However, the cell population of annexin V (+)/PI (+) in the Lf-GL treatment group was 0.28%, which was even lower level to 1.40% in the Control group


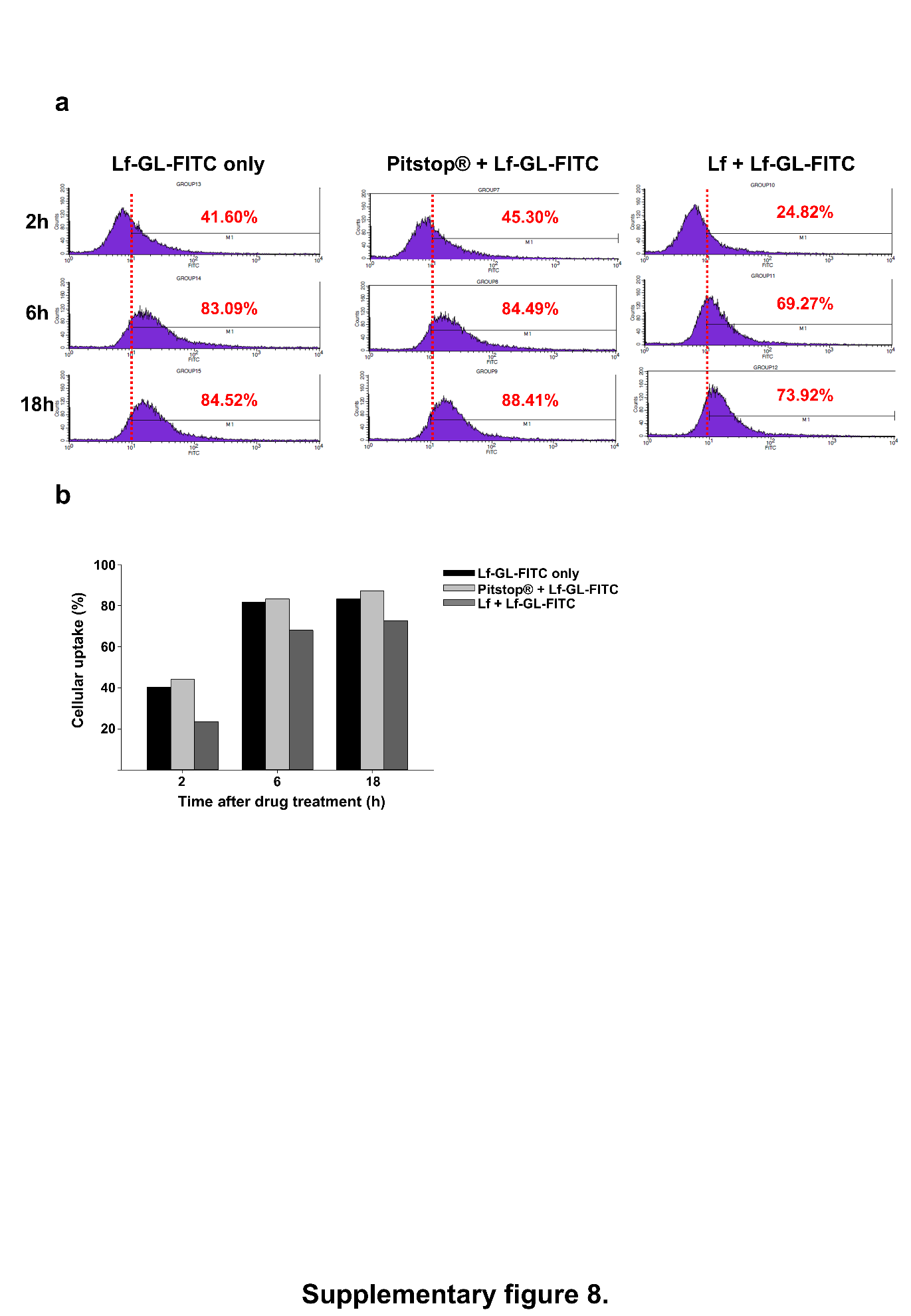


**Fig. S11. Significant cellular uptake of Lf-GL in U87MG.** (a) Flow cytometry to evaluate cellular uptake of FITC tagged-Lf-GL (1 μM) in U87MG for 2,6, and 18 h. The 2 h pre-treatment of Lf and Pitstop® were treated in the concentration of 500 nM and 75 nM, respectively. (b) Quantification of cellular uptake for 18 h. Data were expressed as mean ± S.E.M (n=2).


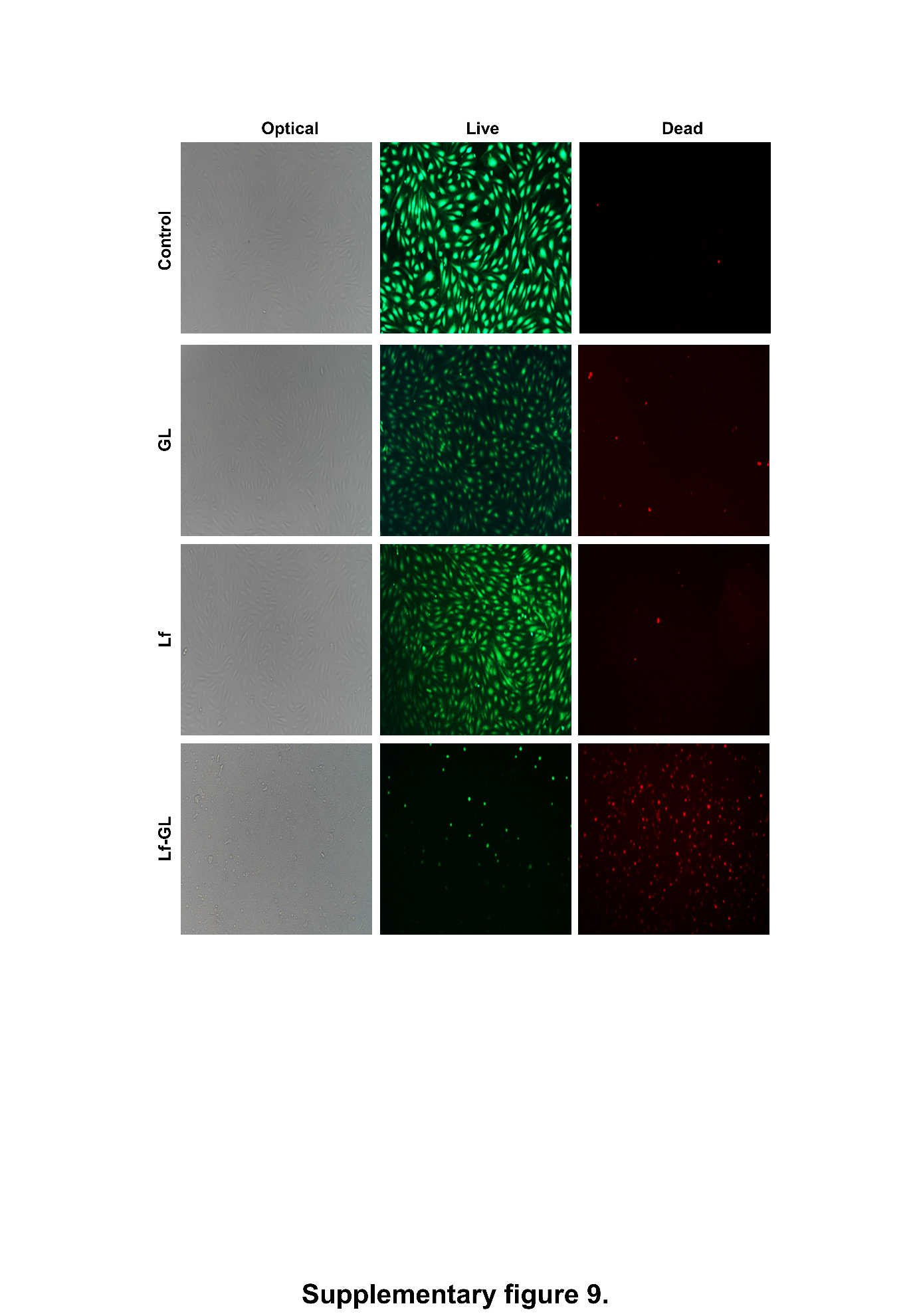


**Fig. S12. Live and dead assay of HUVEC in the Control, GL, Lf, and Lf-GL group.** The cells were treated with GL equivalent concentration of 100 μM. for 24 h. Thereafter, cells were treated with 1 μM of Calcein AM and EthD-1. Magnification: X100


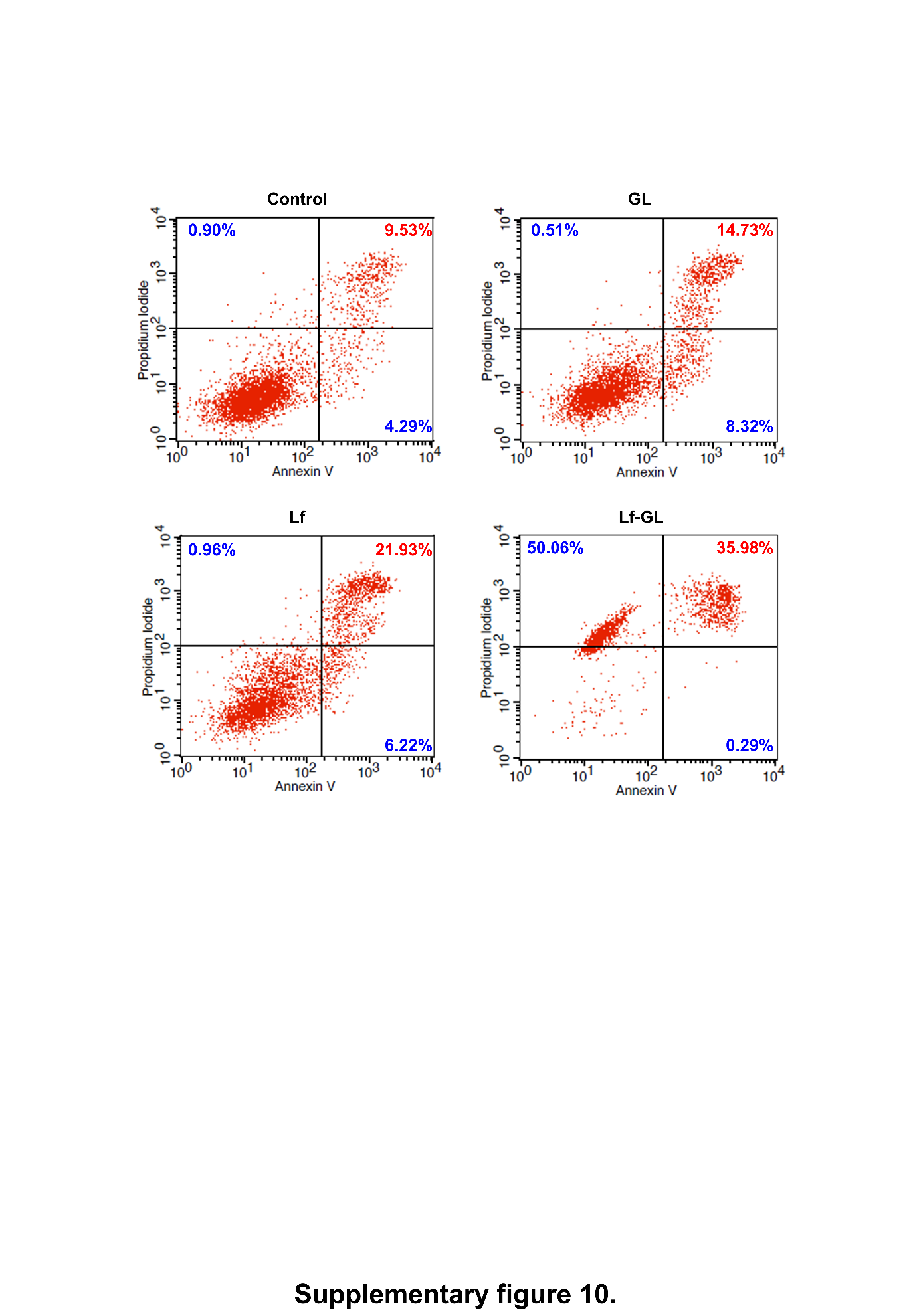


**Fig. S13. HUVEC growth inhibition of Lf-GL.** Annexin V-DY-634/PI apoptosis staining to detect the apoptosis and necrosis in the Control, GL, Lf, and Lf-GL treated group at all GL equivalent concentration of 100 μM for 24 h.


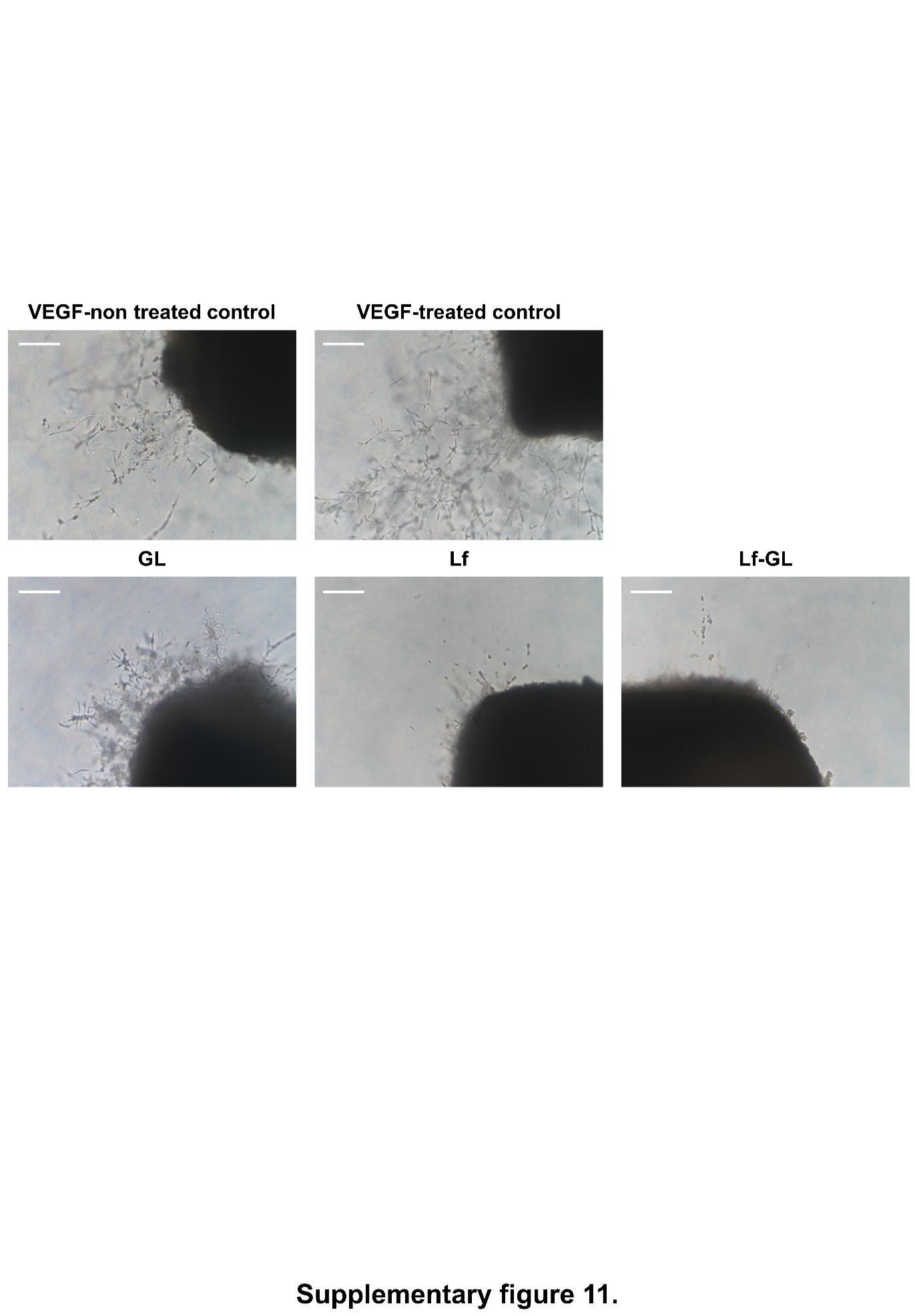


**Fig. S14**. **Significant inhibitory effect of Lf-GL in aortic ring angiogenesis.** Optical images of vessel regression in aortic ring with Lf-GL treatment. Aortic ring assay was carried out in 48-well plate coated with Matrigel. VEGF-non treated control represents that aortic ring incubated with normal medium. VEGF-treated control represents that aortic ring incubated with 25 ng mL^-1^ VEGF-conditioned medium. GL, Lf and Lf-GL groups represents that aortic ring incubated with VEGF-conditioned medium containing GL, Lf, and Lf-GL with GL equivalent concentration of 200 μM, respectively. Magnification: X100. Scale bar: 100 μm.


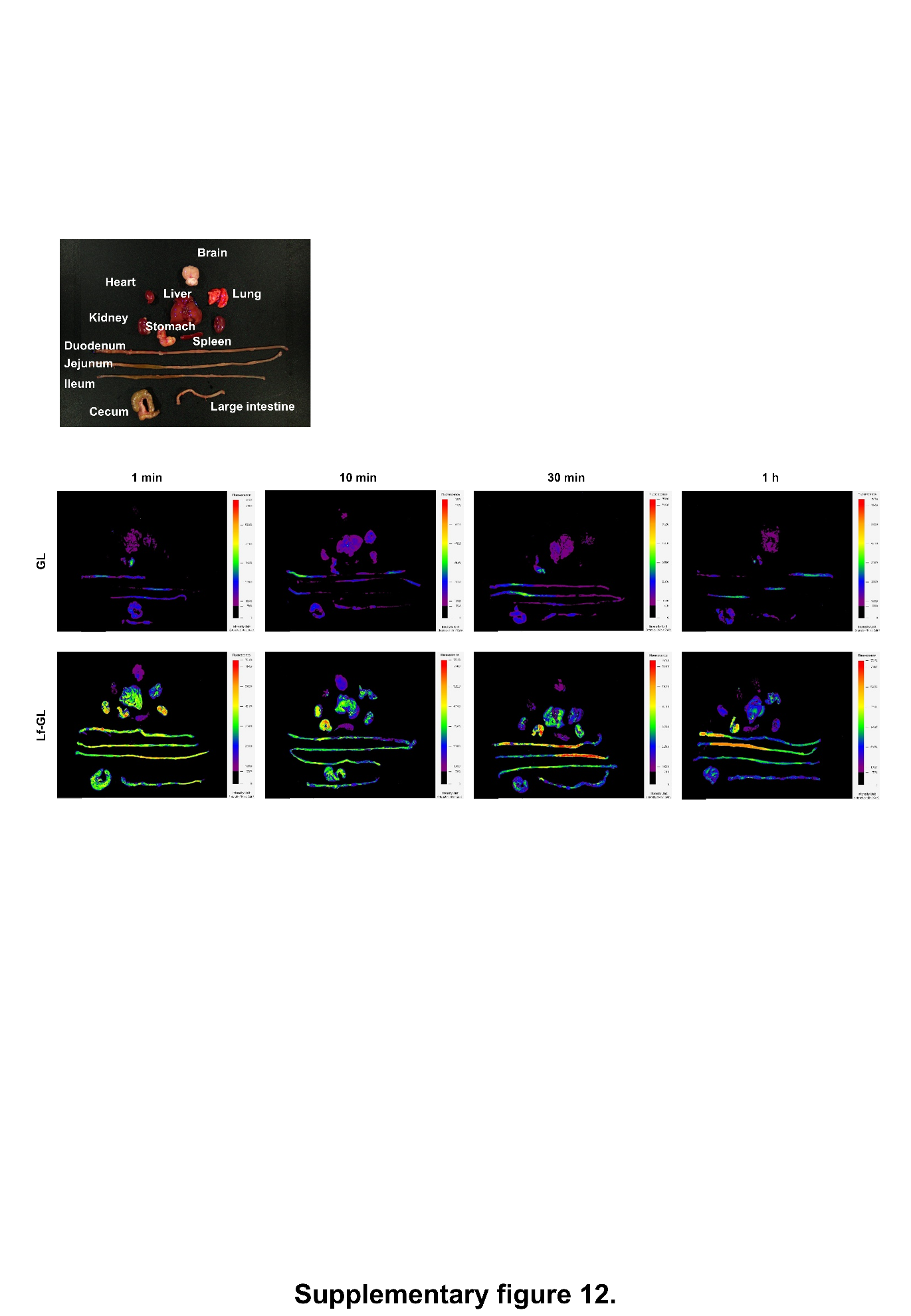


**Fig. S15**. **Fluorescence tracer images of intravenously injected GL and Lf-GL.** Balb/c mice were administered either FITC-tagged GL or FITC-tagged Lf-GL at a GL equivalent concentration of 50 mg kg^-1^ body weight via tail vein injection. The fluorescence signals of FITC-tagged GL or FITC-tagged Lf-GL in organs were imaged using an in vivo imaging system (FOBI, CELLGENTEK, South Korea). The exposure time was fixed to 200 sec for analyzing fluorescent signals from tissues. The quantified fluorescence signal of brain at each time point was measured with Intensity Unit (IU, Intensity Min^-1^ Gain^-1^).


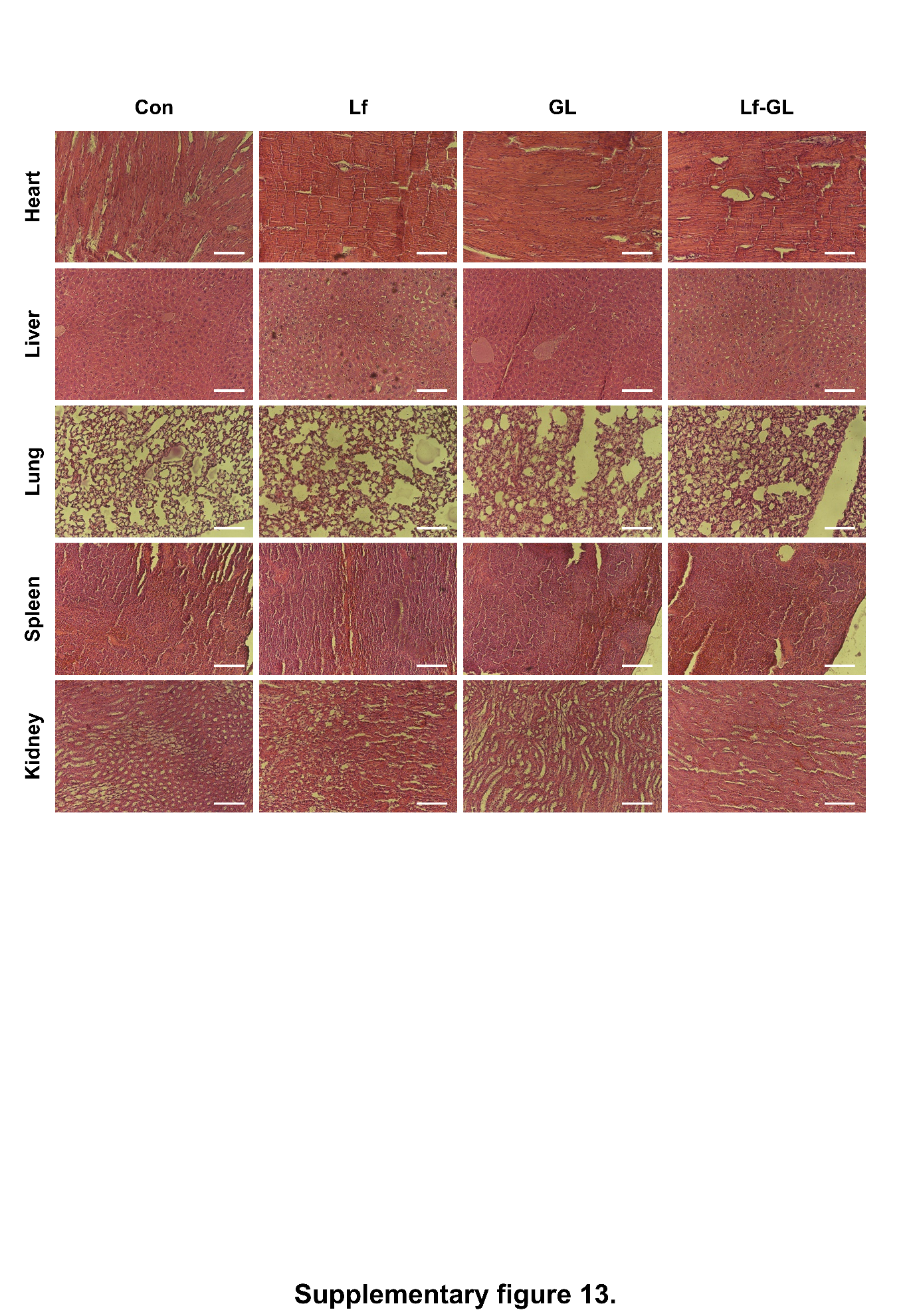


**Fig. S16**. **Histopathological analysis of heart, liver, lung, spleen, and kidney after 28 doses.** The tissues were histologically analyzed after intravenous administration of daily doses (equivalent GL concentration dose of 50 mg kg^-1^ and Lf concentration dose of 5 mg kg^-1^) to GBM-modeled mice for 28 days. Scale bar: 50 μm.
